# Genome-wide DNA methylation analysis of extreme phenotypes in the identification of novel epigenetic modifications in diabetic retinopathy

**DOI:** 10.1186/s13148-022-01354-z

**Published:** 2022-10-31

**Authors:** Shaopeng Yang, Xiao Guo, Weijing Cheng, Ishith Seth, Gabriella Bulloch, Yifan Chen, Xianwen Shang, Zhuoting Zhu, Wenyong Huang, Wei Wang

**Affiliations:** 1 State Key Laboratory of Ophthalmology, Zhongshan Ophthalmic Center, Sun Yat-Sen University, Guangdong Provincial Key Laboratory of Ophthalmology and Visual Science, Guangdong Provincial Clinical Research Center for Ocular Diseases, Guangzhou, China; 2 Centre for Eye Research Australia, Royal Victorian Eye and Ear Hospital, University of Melbourne, Level 7, 32 Gisborne Street, East Melbourne, VIC 3002 Australia; 3 John Radcliffe Hospital, Oxford University Hospitals NHS Foundation Trust, Oxford, UK

**Keywords:** Diabetic retinopathy, Type 2 diabetes mellitus, DNA methylation, Extreme phenotypes, Biomarkers

## Abstract

**Background:**

Aberrant epigenetic modifications such as DNA methylation may contribute to the pathogenesis of DR. We aimed at elucidating the role of novel DNA methylation modifications in diabetic retinopathy (DR) in patients with type 2 diabetes mellitus (T2DM) using an extreme phenotypic design.

**Methods/results:**

Two consecutive studies were conducted. A cross-sectional study using an extreme phenotypic design was conducted to identify rare methylation modifications that might contribute to DR pathogenesis. A 2-year longitudinal nested case–control study was conducted to validate the results and assess whether these novel methylation modifications could be used as biomarkers for predicting DR onset. A large number of differentially methylated CpG sites were identified in the cross-sectional study, and two (cg12869254 and cg04026387) corresponding to known genes were replicated in the longitudinal study. Higher methylation of cg12869254 significantly correlated with macular RNFL thinning in the superior and nasal subregions, and that of cg04026387 correlated with reduced deep capillary plexus VD in the superior and inferior subregions after adjusting for covariates.

**Conclusions:**

Cg12869254 and cg04026387 hypermethylation may complement the known risk factors that contribute to the pathogenesis of DR and as novel biomarkers for disease prediction.

**Supplementary Information:**

The online version contains supplementary material available at 10.1186/s13148-022-01354-z.

## Introduction

Diabetic retinopathy (DR) represents a microvascular end-organ complication of diabetes and is a leading cause of blindness in the working-age population [1]. DR has an insidious onset that delays early detection and treatment [2]. The traditional pathogenesis of DR is largely driven by risk factors such as hyperglycemia, hypertension, and dyslipidemia; however, they explain less than 12% of the risk of developing DR [3]. Growing evidence suggests a clear polygenic risk of developing DR, highlighting the need for new biomarkers for DR. [4]

Aberrant epigenetic modifications such as DNA methylation may contribute to the pathogenesis of DR [5, 6]. Maghbooli et al. [7] noted changes in peripheral blood DNA methylation in DR patients, suggesting the potential value of DNA methylation as a biomarker for DR. However, DNA methylation studies conducted on type 2 diabetes mellitus (T2DM) cohorts based on traditional epigenome-wide association study (EWAS) approaches have yielded unclear results, failing to identify epigenetic modifications reproducibly associated with DR [8–10]. The results of these studies were skewed due to the incomplete phenotypes of the patients included. Moreover, the traditional EWAS approaches used in previous studies are inherently limited to common epigenetic modifications, which means that rare changes with large effects will be missed. Extreme phenotypic design is an emerging study design with superior power for detecting rare variants and has been suggested for the screening and identification of DR markers [11–15]. Further, previous studies followed a cross-sectional design and lack of optical coherence tomography (OCT) and OCTA (OCT angiography) biomarkers, which limits causal inferences and interpretations of their results [8–10]. Lastly, existing studies did not collect patients’ systemic and ocular factors, specifically renal function and axial length (AL), which are known to be independently associated with DR onset and progression. [16–19]

In the present study, we hypothesized that the use of extreme phenotypic designs would greatly increase the power to identify candidate sites, thus providing the ability to search for rare DNA methylation modifications. In the discovery stage, a large number of differentially methylated CpG sites (DMSs) in DR were identified from a cross-sectional study. A nested case–control study was conducted to validate the results and longitudinally assess whether aberrant methylation can be used as a biomarker to predict DR. To determine whether these candidate sites play a role in DR pathogenesis, the association between the methylation degree of these sites with macular retinal nerve fiber layer (RNFL) thickness and vessel density (VD) was analyzed. Since diabetic kidney disease often develops simultaneously with DR, we also assessed the potential association of these sites with renal function. [20]

## Methods

### Study participants

This cross-sectional and longitudinal nested case–control study was conducted at Zhongshan Ophthalmic Center (ZOC) in Guangzhou, China. The study protocol was approved by the institutional review board of ZOC, Sun Yat-sen University (2017KYPJ094). The study procedure followed the principles of the Declaration of Helsinki, and written informed consent was obtained from all the enrolled participants.

Forty-three T2DM patients with DR and 92 T2DM patients without DR at baseline were recruited from 2017 to 2019. All patients were diagnosed with T2DM according to the criteria of the American Diabetes Association [21]. DR was defined according to the revised Early Treatment Diabetic Retinopathy Study (ETDRS) grading scale based on 7-field retinal photography and OCT/A imaging. Patients were excluded from the study if they had (1) severe systemic diseases other than T2DM; (2) cognitive impairment, mental illness, or inability to cooperate with questionnaires or examinations; (3) best-corrected visual acuity (BCVA) less than 20/25; (4) intraocular pressure (IOP) greater than 21 mm Hg; (5) spherical equivalent greater than +3.0 diopters (D) or less than -6.0 D; (6) glaucoma, age-related macular degeneration, amblyopia, and other eye diseases; (7) history of previous intraocular surgery or any other ophthalmic treatment; (8) pterygium and severe refractive media clouding.

Among these patients, 10 were DR-free despite > 20 years of T2DM. All 92 patients without DR at baseline were followed up for 2 years, and 24 of them had new-onset DR. Two consecutive studies were conducted. The cross-sectional study serves as the discovery stage, and the results were then validated in the longitudinal study. The cross-sectional study included (1) a control group (*n* = 10) diagnosed with T2DM for at least 20 years without any clinical signs or visual-related symptoms of DR, referred to as the NDR group, and (2) a case group (*n* = 10) of patients diagnosed with DR within 4 years of T2DM diagnosis matched 1:1 by age, sex, and glycated hemoglobin (HbA1c), referred to as the DR group. The longitudinal study enrolled patients without DR at baseline within 2 years of T2DM diagnosis and divided them into two groups according to DR outcome during the 2-year follow-up: (1) DR high-risk group (*n* = 10): patients with DR onset within 2 years, referred to as the incidence-DR group; and (2) DR stable group (*n* = 10): patients who remained NDR during the 2-year period matched similarly to the incidence-DR group, referred to as the stable-NDR group (Additional file 2: Fig. S1).

### Clinical examinations and SS-OCT/A image acquisition protocol

We obtained a 2 mL fasting venous blood sample anticoagulated with disodium ethylenediaminetetraacetate from each participant and stored at − 80 °C without repeated freezing and thawing. Questionnaires and systemic and ocular examinations were administered to all participants. Details are as follows. After dilatation, seven fundus photographs were obtained according to the ETDRS-7 protocol, using a digital fundus camera (Canon CX-1, Tokyo, Japan). All participants completed a standardized questionnaire to collect information on sex, age, and medical history. Systolic blood pressure, diastolic blood pressure, height (m), and weight (kg) were measured. Body mass index (BMI) was defined as the weight divided by the square of height. Venous blood and urine samples were collected to assay for serum total cholesterol, high-density lipoprotein cholesterol, low-density lipoprotein cholesterol, triglycerides, HbA1c, ACR (albumin creatinine ratio), and estimated glomerular filtration rate (eGFR). The eGFR was calculated using the Cockcroft-Gault formula [22]. The IOP was measured using a non-contact IOP meter (mputeCT-1 Corized Tonometer, Topcon Ltd., Topcon). BCVA was measured using an ETDRS LogMAR visual acuity chart (Precision Vision, Villa Park, Illinois, USA). The AL was measured using a Lenstar LS 900 biometer (HAAG-Streit AG, Köniz, Switzerland).

A commercial SS-OCT imaging system (Triton; Topcon Inc., Tokyo, Japan) was used to obtain structural OCT and OCTA images. The instrument has axial and lateral resolutions of 8 and 20 µm, respectively, and uses a wavelength of 1050 nm with a scan speed of 100,000 A-scans per second. Transverse section images of the fundus and en-face images of the retinal microvascular systems were obtained using a 3D Macula Cube 7 × 7 mm scan mode with a scan density of 512 A-scans × 512 B-scans and Angio Macula 3 × 3 mm scan mode with a scan density of 320 A-scans × 320 B-scans, both centered on the fovea. The transverse section images were automatically segmented into multiple layers, and measurements of the RNFL thickness, including measurements of the superior, inferior, nasal, and temporal regions, were obtained. The en-face images of the retinal vascular systems were automatically segmented into four slabs, including the superficial capillary plexus (SCP) and deep capillary plexus (DCP), and the VDs of each subregion (superior, inferior, nasal, and temporal) in both SCP and DCP slabs were obtained. The results of the automated segmentation were evaluated by an image expert, and the participants’ data were masked during processing. Manual adjustments were performed if segmentation errors were present.

### Genome-wide DNA methylation analysis

Whole blood DNA was extracted from fasting venous blood samples using the DNeasy Blood and Tissue Kit (Qiagen, Hilden, Germany). The purity and concentration of the DNA were estimated using a NanoDrop 2000 spectrophotometer (Thermo Scientific). Qualified samples were stored at -20 degrees. Approximately 500 ng of genomic DNA from each sample was used for sodium bisulfite conversion using the EZ DNA methylation Gold Kit (Zymo Research, USA), following the manufacturer’s standard protocol. An Infinium MethylationEPIC BeadChip Kit (Illumina Inc., USA) was used to perform genome-wide DNA methylation analyses. The distribution of cell types was inferred using the robust partial correlation method based on DNA methylation signatures of the constituent cell types.

Probe filtering was performed according to the manufacturer’s protocol to ensure valid analysis. Specifically, all (1) probes with detection *p* values ≥ 0.01, (2) probes with a bead count less than 3 in more than 5% of samples, (3) non-CpG probes, (4) multi-hit probes, (5) SNP-related probes (within 5 bp of an SNP), and (6) probes located in X and Y chromosomes were filtered out.

The experimental quality of each stage of the genome-wide methylation analysis was carefully assessed using internal reference samples. Staining controls were used to examine the sensitivity and efficiency of the X-staining stage. The efficiency of single-base extension at the X-staining stage was examined using extension controls. Hybridization controls were used to examine the efficiency of DNA hybridization. Target removal controls were used to determine the efficiency of stripping off DNA templates after the single-base extension phase. Bisulfite conversion controls were used to evaluate the conversion efficiency of sulfite to genomic DNA. Specific controls were used to detect non-specific extensions of Infinium I and Infinium II probes. The overall performance of the assay and the sample quality were assessed using non-polymorphic controls. Finally, random sequences that did not hybridize with the target DNA were used as negative controls. The *β* value was calculated and normalized for quantifying the degree of methylation (Additional file 1: Methods).

### Statistical analyses

R software (V4.1.0) was used for all data analyses and the presentation of the results. Continuous variables were presented as means ± standard deviations (SD), and categorical variables were presented as counts (proportions). Continuous variables were compared using Student’s *t* test, and categorical variables were compared using chi-square tests. To derive methylation levels, array data were analyzed using the ChAMP package (V2.14.0). Batch effects were corrected using the ComBat method. The limma package (V3.40.6) was used to perform linear regression and modified *t* tests, with age, sex, serum creatinine, AL, and cellular composition included as covariates. Sites that met the significance level with a |Δ*β*|≥ 0.1 were used for further analyses. Gene Ontology (GO) and Kyoto Encyclopedia of Genes and Genomes (KEGG) enrichment analyses were performed to analyze the function of differentiated genes. The Benjamini–Hochberg method was used to reduce the false discovery rate. The association between the degree of methylation of the identified sites and OCT/A measurements, renal function, and metabolic indexes was analyzed using multivariate linear regression. Two-sided p values less than 0.05 were considered statistically significant.

## Results

### Characteristics of the study groups

The demographics and characteristics of the subjects are summarized in Table 1. In the cross-sectional analysis, subjects from the DR and matched NDR groups shared similar characteristics, with the exception of BCVA (*P* = 0.007). Similarly, none of the systemic or ocular factors showed any significant differences between the incidence-DR and matched stable-DR groups in the prospective analysis (all *P* > 0.05). This indicated that the baseline characteristics of the control and case groups were well matched. The baseline characteristics of patients in the incidence-DR group in the prospective analysis were equally comparable to those of patients in the DR group in the cross-sectional analysis (all *P* > 0.05) (Additional file 2: Table S1).

**Table 1:** Demographic and ocular characteristics of the study population

Characteristics	Cross-sectional study		*P* value*	Longitudinal study		*P* value
	NDR group	DR group		Stable-NDR group	Incidence-DR group	
Number of subjects	10	10	–	10	10	–
Age, year	61.4 ± 3.2	64.2 ± 3.3	0.071	67.3 ± 10.3	66.3 ± 6.4	0.798
Male, %	5 (50%)	6 (60%)	0.653	3 (30%)	3 (30%)	1.000
BMI, kg/m^2^	26.0 ± 2.9	24.3 ± 2.5	0.194	24.2 ± 1.4	26.3 ± 4.2	0.152
SBP, mm Hg	143.4 ± 15.0	139.4 ± 22.5	0.646	135.9 ± 10.9	139.1 ± 19.4	0.654
DBP, mm Hg	73.2 ± 5.6	79.3 ± 13.9	0.214	72.9 ± 8.0	70.1 ± 9.5	0.486
HbA1c, %	7.4 ± 1.5	6.8 ± 1.5	0.415	6.4 ± 0.7	6.9 ± 0.3	0.125
TG, mmol/L	2.2 ± 1.0	2.7 ± 2.2	0.476	2.1 ± 1.1	2.1 ± 0.9	0.951
TC, mmol/L	4.8 ± 0.7	4.7 ± 0.7	0.874	4.4 ± 1.1	4.8 ± 0.6	0.363
LDL-c, mmol/L	3.0 ± 0.7	2.9 ± 0.8	0.762	2.9 ± 1.2	3.1 ± 0.8	0.611
HDL-c, mmol/L	1.3 ± 0.4	1.2 ± 0.4	0.481	1.2 ± 0.3	1.5 ± 0.3	0.059
Creatinine, μmol/L	69.5 ± 13.4	77.1 ± 21.6	0.357	66.4 ± 13.5	71.0 ± 14.3	0.480
ACR, mg/g	6.7 ± 0.7	10.5 ± 2.1	0.100	8.7 ± 5.5	7.3 ± 1.2	0.494
eGFR, mL/min †	92.5 ± 20.7	75.2 ± 19.2	0.078	74.8 ± 6.7	78.6 ± 6.2	0.686
BCVA, logMAR	0.3 ± 0.1	0.2 ± 0.1	**0.007**	0.2 ± 0.1	0.2 ± 0.2	0.883
AL, mm	23.7 ± 0.6	23.5 ± 1.5	0.594	23.4 ± 1.1	23.3 ± 0.7	0.803
IOP, mmHg	16.2 ± 2.5	15.7 ± 2.7	0.665	14.1 ± 3.1	15.2 ± 2.3	0.397
T2DM duration, year	22.0 ± 0.7	2.2 ± 0.4	< **0.001**	2.4 ± 0.4	2.3 ± 0.5	0.930
Insulin, %	3 (30%)	1 (10%)	0.303	1 (10%)	1 (10%)	1.000
Oral antihyperglycemic agents, %	10 (100%)	10 (100%)	1.000	10 (100%)	10 (100%)	1.000

Data are expressed as the mean ± standard deviation or n (%). BMI = Body mass index; SBP = systolic blood pressure; DBP = diastolic blood pressure; HbA1c = glycosylated hemoglobin; TG = triglycerides; TC = total cholesterol; LDL-c = low-density lipoprotein cholesterol; HDL-c = high-density lipoprotein cholesterol; ACR = albumin creatinine ratio; eGFR = estimated glomerular filtration rate; BCVA = best-corrected visual acuity; AL = axial length; IOP = intraocular pressure; DM = diabetic mellitus; DR = diabetic retinopathy

*Bold indicates statistically significant

†The eGFR was calculated using the Cockcroft-Gault formula

### Genome-wide DNA methylation analysis

**Fig. 1:**
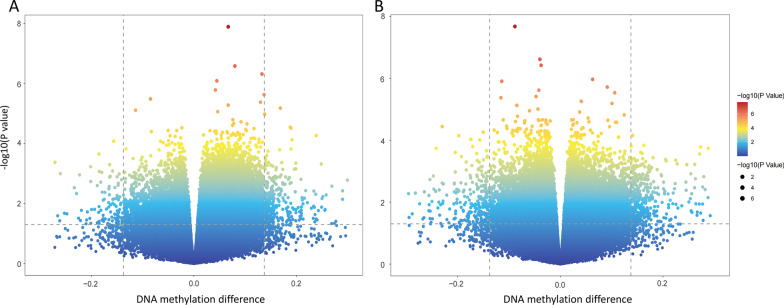
Volcano plot for differential DNA methylation analysis of **A** DR group and NDR group, and **B** incidence-DR group and stable-DR group. DR: diabetic retinopathy; NDR: non-DR

**Fig. 2:**
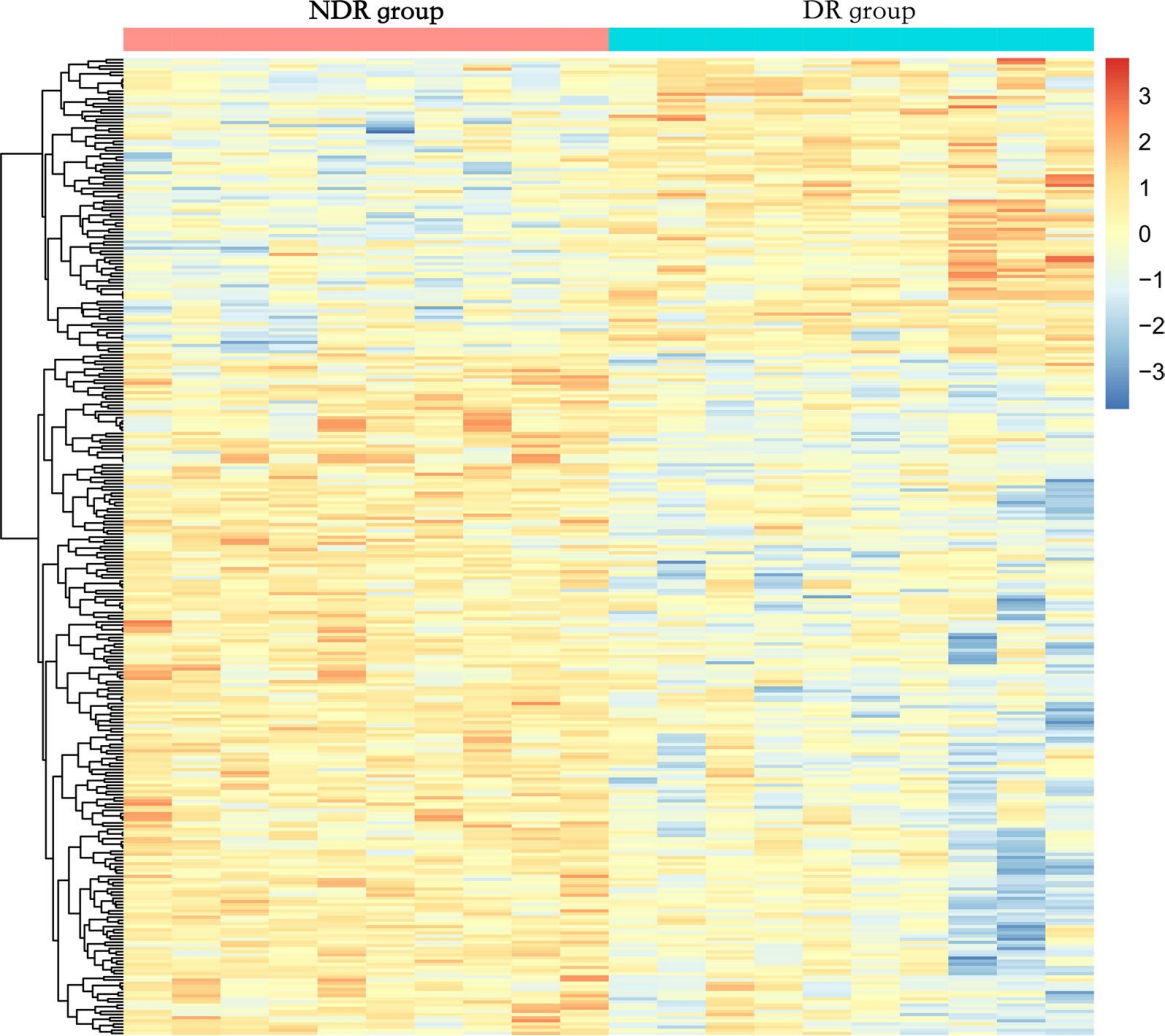
Heat map illustrating differentially methylated sites of DR group and NDR group. Red and blue represent levels of hypermethylation and hypomethylation. DR: diabetic retinopathy; NDR: non-DR

Comparisons revealed significant differences in cellular composition, both in cross-sectional and in longitudinal studies (*P* < 0.05) (Additional file 2: Figs. S2–S5). The DNA quality was adequate with values of 1.8–2.0 for optical density (OD) 260/OD280. The whole analysis was reliable given that all analytical stages passed rigorous quality control (Additional file 2: Figs. S6–S13). The methylation profiles were compared separately in two comparison groups: DR group versus NDR group, and incidence-DR group versus stable-NDR group. A total of 311 and 413 novel DMSs were identified in the cross-sectional and longitudinal studies, respectively (Fig. 1a, b). The DMSs identified in the cross-sectional study were contained in 141 genes, including CACNA1C, COL19A1, FLT3, S100A13, ZDHHC23, and SLC25A21. The ten most significantly differentiated genes between the NDR and DR groups are listed in Table 2. Compared to the NDR group, 217 sites (69.8%) were hypomethylated and 94 sites (30.2%) were hypermethylated (Fig. 2). Among them, 59 sites were located in the promoter region, 112 in the body region, 7 in the 3′-untranslated region (3′-UTR), 1 in the ExonBnd region, and 32 in the intergenic region (IGR). Concerning CpG positions, 43 sites were located in CpG islands, 183 in the open sea, 10 in the shelf regions, and 75 in the shore regions (Additional file 2: Fig. S14A, B).

**Table 2:** Top 10 significant genes harboring differentially methylated CpG positions (DMPs) between NDR group and DR group

DMS	Gene	CHR	FGD	Cgi	*β* value			*P* value
					NDR group	DR group	Difference	
cg05129572	NEDD4	15	Body	Open sea	0.596	0.430	− 0.166	5.41 × 10^−5^
cg12507788	MRPL4	19	TSS1500	Shore	0.759	0.627	− 0.133	7.48 × 10^–5^
cg07469467	APAF1	12	Body	Open sea	0.381	0.140	− 0.241	1.52 × 10^–4^
cg27535148	COL19A1	6	Body	Open sea	0.381	0.265	− 0.116	1.60 × 10^–4^
cg06773116	KCNIP4	4	Body	Open sea	0.503	0.631	0.128	2.2 3 × 10^–4^
cg13563074	OVCH2	11	TSS1500	Open sea	0.498	0.397	− 0.101	4.22 × 10^–4^
cg02294028	MDFIC	7	Body	Shore	0.427	0.308	− 0.119	4.26 × 10^–4^
cg24063962	LOC100130274	8	TSS200	Island	0.607	0.721	0.114	5.02 × 10^–4^
cg02738594	CAMK1G	1	Body	Open sea	0.456	0.596	0.140	5.86 × 10^–4^
cg11686436	FAM83E	19	Body	Shore	0.824	0.713	− 0.112	6.33 × 10^–4^

DMP = differentially methylated CpG position; CHR = chromosome; FGD = functional genomic distribution; Cgi = CpG island; NDR = without any diabetic retinopathy; DR = diabetic retinopathy

**Fig. 3:**
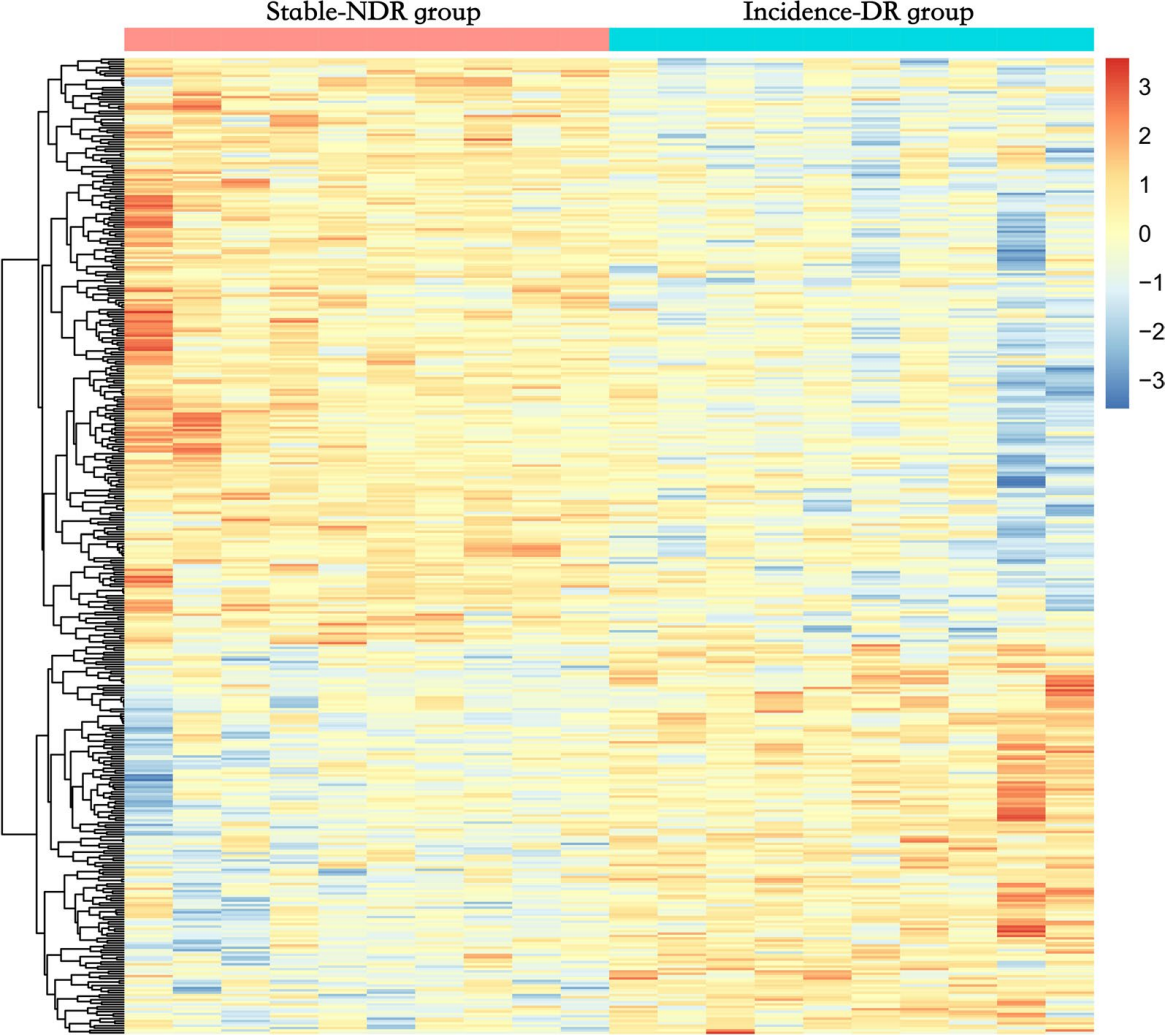
Heat map illustrating differentially methylated sites of incident-DR group and stable-NDR group. Red and blue represent levels of hypermethylation and hypomethylation. DR: diabetic retinopathy; NDR: non-DR

The longitudinal nested case–control study identified 197 novel genes, including MICAL1, NLGN1, CACNG2, ZDHHC23, and SLC25A21. The ten most significantly differentiated genes between the stable-NDR and incidence-DR groups are listed in Table 3. Compared to the stable-NDR group, 248 sites (60.0%) were hypomethylated and 165 sites (40.0%) were hypermethylated (Fig. 3). Further analysis of the DNA functional domains revealed that 102 sites were located in the promoter region, 151 in the body region, 6 in the 3’-UTR, 1 in the ExonBnd region, and 153 in the IGR. Regarding CpG positions, 62 sites were located in CpG islands, 219 in the open sea, 15 in the shelf regions, and 117 in the shore regions (Additional file 2: Fig. S14C, D). The results of GO and KEGG pathway analyses are shown in Additional file 2: Figs. S15 and S16.

**Table 3:** Top 10 significant genes harboring DMP between stable-NDR group and incidence-DR group

DMS	Gene	CHR	FGD	Cgi	*β* value			*P* value
					Stable-NDR group	Incident-DR group	Difference	
cg05119883	ZMIZ1-AS1	10	Body	Open sea	0.539	0.324	− 0.215	2.61 × 10^−5^
cg04145948	BLM	15	Body	Open sea	0.357	0.516	0.159	3.63 × 10^−5^
cg19522093	PPP5D1	19	Body	Open sea	0.111	0.215	0.104	5.48 × 10^−5^
cg10725855	PCDHB12	5	1stExon	Shore	0.679	0.816	0.137	7.14 × 10^−5^
cg17682313	FBXW7	4	5'UTR	Open sea	0.768	0.209	− 0.559	1.09 × 10^−4^
cg17457090	NDRG4	16	TSS200	Shore	0.439	0.250	− 0.189	1.66 × 10^−4^
cg17666981	TRIM27	6	Body	Open sea	0.639	0.806	0.167	1.82 × 10^−4^
cg24093474	NDRG4	16	TSS1500	Shore	0.715	0.515	− 0.200	1.82 × 10^−4^
cg17107388	NDRG4	16	TSS1500	Shore	0.710	0.445	− 0.264	1.86 × 10^−4^
cg01877814	POLG	15	TSS1500	Shore	0.327	0.218	− 0.109	2.45 × 10^−4^

DMP = differentially methylated CpG position; CHR = chromosome; FGD = functional genomic distribution; Cgi = CpG island; NDR = without any diabetic retinopathy; DR = diabetic retinopathy

### Replicated DMSs in the longitudinal study

The two novel DMSs corresponding to known genes identified in the cross-sectional analysis were replicated in the longitudinal analysis. Cg12869254 and cg04026387, contained in the ZDHHC23 and SLC25A21 genes, were the two novel sites that showed significant methylation changes in the same direction in both cross-sectional (cg12869254: *β* difference = + 0.11, *P* = 0.012; cg04026387: *β* difference = + 0.11, *P* = 0.018) and longitudinal analyses (cg12869254: *β* difference = + 0.13, *P* = 0.015; cg04026387: *β* difference = + 0.10, *P* = 0.020). These two sites are both located in the body region concerned with functional genomic distribution and in the open sea relative to CpG islands. Methylation modifications of these two novel DMSs have not been identified using previous traditional GWAS approaches. Hypermethylation at these two sites may serve as a new biomarker for predicting DR.

### Association of identified DMSs with OCT/A measurements

In the univariate linear model, the degree of methylation of cg12869254 was negatively correlated with macular RNFL thickness in the superior (*β* = − 2.6, 95%CI − 5.1, − 0.2; *P* = 0.039) and nasal subregions (*β* = − 2.6, 95%CI − 4.4, − 0.7; *P* = 0.009). The correlation remained stable in the multivariate model after adjusting for age, sex, T2DM duration, and HbA1c level (*β* = − 3.0, 95%CI − 5.8, − 0.2; *P* = 0.035 and *β* = − 2.2, 95%CI − 4.4, − 0.1; *P* = 0.049) (Table 4). The degree of methylation of cg04026387 was found to be negatively correlated with macular DCP VD in the superior (*β* = − 0.8, 95% CI − 1.6, − 0.1; *P* = 0.049) and inferior subregions (*β* = − 1.4, 95% CI − 2.4, − 0.4; *P* = 0.008) and positively correlated with SCP VD in the nasal subregion (*β* = 1.9, 95% CI 0.5, 3.3; *P* = 0.011) in the univariate linear model. The correlation remained stable in model adjusting for age, sex, T2DM duration, and HbA1c (*β* = − 0.8, 95% CI − 1.7, − 0.1; *P* = 0.039, *β* = − 1.5, 95% CI − 2.8, − 0.2; *P* = 0.028, and *β* = 2.2, 95% CI 1.1, 3.3; *P* = 0.001) (Table 5).

**Table 4:** Association between cg12869254 with OCT/A measurements in univariable and multivariable linear regression models

OCT/A measurements*	Univariable model 1			Multivariable model 2^§^		
	*β*	95% CI	*P* value†	*β*	95% CI	P value^†^
SCP VD, %						
Superior	0.4	(− 1.6, 2.2)	0.695	− 0.2	(− 2.5, 2.0)	0.828
Inferior	− 0.6	(− 3.0, 1.7)	0.582	− 0.3	(− 3.5, 2.9)	0.839
Nasal	1.4	(− 0.6, 3.3)	0.164	1.3	(1.0, 3.6)	0.241
Temporal	0.01	(− 2.7, 2.7)	0.990	− 1.7	(− 5.3, 1.9)	0.321
DCP VD, %						
Superior	0.1	(− 1.0, 1.1)	0.895	0.2	(− 1.1, 1.5)	0.728
Inferior	− 0.7	(− 2.1, 0.7)	0.322	0.1	(− 2.0, 2.2)	0.917
Nasal	− 0.2	(− 1.3, 0.9)	0.702	0.2	(− 1.1, 1.5)	0.806
Temporal	− 0.8	(− 2.5, 0.9)	0.334	− 0.7	(− 2.7, 1.3)	0.444
RNFL thickness, µm						
Superior	− 2.6	(− 5.1, − 0.2)	**0.039**	− 3.0	(− 5.8, − 0.2)	**0.035**
Inferior	− 0.4	(− 2.4, 1.6)	0.666	0.3	(− 2.4, 3.0)	0.790
Nasal	− 2.6	(− 4.4, − 0.7)	**0.009**	− 2.2	(− 4.4, − 0.1)	**0.049**
Temporal	0.2	(− 2.0, 2.4)	0.843	0.4	(− 2.1, 2.8)	0.749

OCT/A = optical coherence tomography/angiography; SCP = superficial capillary plexus; VD = vessel density; DCP = deep capillary plexus; RNFL = retinal nerve fiber layer; CI = confidential interval; T2DM = type 2 diabetes mellitus; HbA1c = glycated hemoglobin

^§^Adjusted for age, sex, T2DM duration and HbA1c. ^*^Per percent of methylation change. ^†^Bold indicates statistically significant

**Table 5:** Association between cg04026387 with OCT/A measurements in univariable and multivariable linear regression models

OCT/A measurements*	Univariable model 1			Multivariable model 2^§^		
	β	95% CI	*P* value†	*β*	95% CI	*P* value^†^
SCP VD, %						
Superior	1.4	(− 0.1, 2.8)	0.060	1.0	(− 0.5, 2.5)	0.170
Inferior	− 0.3	(− 2.2, 1.6)	0.769	0.5	(− 1.7, 2.7)	0.650
Nasal	1.9	(0.5, 3.3)	**0.011**	2.2	(1.1, 3.3)	**0.001**
Temporal	0.6	(− 1.6, 2.8)	0.567	1.0	(− 1.5, 3.6)	0.404
DCP VD, %						
Superior	− 0.8	(− 1.6, − 0.1)	**0.049**	− 0.8	(− 1.7, − 0.1)	**0.039**
Inferior	− 1.4	(− 2.4, − 0.4)	**0.008**	− 1.5	(− 2.8, − 0.2)	**0.028**
Nasal	0.2	(− 0.7, 1.1)	0.682	0.5	(− 0.3, 1.4)	0.203
Temporal	− 0.3	(− 1.7, 1.1)	0.698	− 0.5	(− 1.9, 0.8)	0.428
RNFL thickness, µm						
Superior	− 0.1	(− 2.5, 2.3)	0.991	− 0.8	(− 3.2, 1.4)	0.431
Inferior	− 1.1	(− 2.6, 0.5)	0.172	0.1	(− 1.8, 2.0)	0.919
Nasal	− 0.6	(− 2.4, 1.2)	0.491	− 0.2	(− 2.0, 1.6)	0.809
Temporal	0.9	(− 0.8, 2.7)	0.261	1.0	(− 0.6, 2.6)	0.190

OCT/A =optical coherence tomography/angiography; SCP = superficial capillary plexus; VD = vessel density; DCP = deep capillary plexus; RNFL = retinal nerve fiber layer; CI = confidential interval; T2DM = type 2 diabetes mellitus; HbA1c = glycated hemoglobin

^§^Adjusted for age, sex, T2DM duration and HbA1c. ^*^Per percent of methylation change. ^†^Bold indicates statistically significant

### Association of identified DMSs with renal function and metabolic indexes

The degree of methylation of cg12869254 was significantly correlated with reduced eGFR (*β* = − 0.2, 95% CI − 0.4,-0.01; *P* = 0.038), and that of cg04026387 was significantly correlated with elevated ACR (*β* = 0.7, 95% CI 0.2, 1.1; *P* = 0.014), after adjusting for age, sex, T2DM duration, and HbA1c level (Additional file 2: Tables S2 and S3). Several metabolic indexes were also related to the methylation of these two sites (Additional file 2: Table S4**,** Additional file 1: Results).

## Discussion

This study consisted of genome-wide DNA methylation analyses of extreme phenotypes within two consecutive studies that identified novel epigenetic modifications of DR. In the discovery stage, a large number of novel candidate sites were identified. To validate the results and assess whether these methylation modifications could be used as biomarkers to predict DR, we analyzed DNA methylation in an independent longitudinal study. Our results suggest that aberrant methylation may predict DR, as hypermethylation at cg04026387 and cg12869254 identified in the cross-sectional study was successfully replicated in the longitudinal study. Finally, the methylation degree of these two sites negatively correlated with macular RNFL thickness and DCP VD, which further confirmed their potential for indicating DR [23, 24]. Therefore, methylation at these two sites may contribute to neural and vascular damage in the early stages of DR and serve as novel biomarkers for predicting the disease.

In this study, an extreme phenotypic design was adopted to identify the specific DMSs, which enabled us to identify novel methylation modifications, despite the relatively small yet sufficient sample size, as in studies of similar design [25–28]. Conversely, the results of previous studies on DR were skewed by the incomplete phenotypes of included participants, that is, healthy individuals and/or inclusion of individuals with diabetes diagnosis of < 20 years as DR control groups, and/or DR patients with random DR-free T2DM duration as the DR case group. Current evidence suggests that an extreme phenotypic design is necessary for screening and identifying DR markers [29, 30]. The extreme phenotypic design is a recent concept that is used to increase homogeneity and better distinguish signals from noise in genome-wide analysis. Under this approach, the decreased variation in homogeneous samples results in a robust ability to identify signals, even in a relatively small number of samples. Concurrently, it enables the discovery of rare methylation alternations, which is another advantage of this design. Thus, we included patients diagnosed with T2DM for at least 20 years without any signs of DR as an extreme control group, and patients diagnosed with DR within 4 years of T2DM diagnosis were included as an extreme case group. As most patients typically develop DR within 17 years of T2DM diagnosis [11], while epigenetic changes typically initiate retinal impairment within a short period, the enrolled patients may represent extreme phenotypes enriched in “protective” and “risk” epigenetic alternations, respectively. Therefore, this model increased the candidate site identification in this study.

To our knowledge, this is the first study to longitudinally investigate the relationship between DNA methylation and T2DM in patients with DR. Thus far, all previous studies on T2DM patients have been cross-sectional, which limits causal inferences of their results. However, DNA methylation has been suggested to be dynamic rather than rigidly fixed in various diseases and aging process [31]. Therefore, it is important to clarify the time-ordered relationships between such aberrant methylation and DR pathogenesis. Our cross-sectional study revealed significant hypomethylation in sites within S100A13 in the gene-positive DR group, which is in agreement with the results obtained by Li et al. [8] Demethylation of this gene purportedly increases hyperglycemia-induced damage through calcium signaling and the RAGE pathway. However, these results were not replicated in the longitudinal study. Similarly, many other candidate sites identified in the cross-sectional discovery stage exhibited no significant differences in the longitudinal study. This highlights the limitations of previous cross-sectional studies in which the chronological relationship between exposure and outcome was unclear. The changes in methylation at these sites may be explained as concomitant consequences of DR development rather than as primary contributing factors to DR pathogenesis or progression. Cg12869254 and cg04026387 were successfully replicated in this longitudinal study, suggesting that methylation alterations at these two sites precede DR, supporting their role in the early pathogenesis of DR.

The two novel sites identified in this study are included in the ZDHHC23 and SLC25A21 genes, which have not been identified in previous studies. A recent animal study demonstrated the neurotoxic effects mediated by the downregulation of the ZDHHC23 gene [32]. Decreased ZDHHC23 levels dysregulated interactions between palmitoyl-protein thioesterase-1 and acyl protein thioesterase-1, leading to increased plasma membrane H-Ras and subsequent microglial proliferation in mouse brains. Activation of microglia leads to increased secretion of tumor necrosis factor-alpha, interleukin-6, monocyte chemoattractant protein-1, and complement component C1q, promoting the transformation of astrocytes to the neurotoxic A1 phenotype [33]. The astrocytes of the A1 phenotype not only lose their original neurotrophic role, but also actively produce neurotoxins that degrade neurons. Retinal neurodegeneration is considered an early component of DR, which can precede visible vasculopathy [23, 24]. In this study, the degree of methylation of cg12869254 negatively correlated with macular RNFL thickness in the superior and nasal subregions, while the RNFL thickness represented the axons of retinal ganglion cells. Our teams and other groups have demonstrated that reduced RNFL thickness was significantly associated with a higher risk of development and progression of DR [34]. Therefore, we hypothesize that ZDHHC23 gene-mediated neurotoxicity caused by hypermethylation of cg12869254 may also contribute to the retinal neurodegeneration in DR. Additional studies are warranted to further elucidate the potential role of ZDHHC23 in DR.

SLC25A21 is involved in intracellular organic acid catabolism and metabolism [35]. Within mitochondria, it transports 2-oxoadipate and 2-oxoglutarate to be converted into acetyl-CoA. Boczonadi et al. [36] found that the dysfunction of SLC25A21 may lead to impaired transport of 2-oxoadipate, which in turn disrupts the degradation of lysine and tryptophan, leading to the accumulation of 2-oxoadipate, piperidinic acid, and quinolinic acid. Overexposure to these cytotoxic substances leads to irreversible mitochondrial and tissue damage mediated by free radicals. Consistently, the methylation degree of cg04026387 locus negatively correlated with macular DCP VD in our study, suggesting that it is a more sensitive indicator than VD in other plexus [37]. Therefore, we hypothesize that similar mechanisms of mitochondrial damage and vascular damage may also occur in the pathogenesis of early DR. However, SCP VD in the nasal subregion appears to be increased with higher methylation of cg04026387. Similar results have been reciprocated in studies on rats and humans [38, 39]. One possible explanation for this is that the deeper capillary layer is more sensitive to mitochondrial damage. In the early stages of DR, the above-mentioned factors may act mainly on the DCP, whereas the SCP exhibits a compensatory flow increase secondary to the reduced DCP VD. However, further studies investigating these genes are required to validate our hypothesis.

DKD is another major microvascular complication of diabetes mellitus [40]. Given the commonalities in pathogenesis and frequent co-development of DR and DKD, we further analyzed the relationship between these two sites and renal function indexes [20, 41–43]. Hypermethylation at these two identified sites was significantly associated with elevated ACR and reduced eGFR, further increasing our confidence in the involvement of these two sites in diabetic microvascular complications. We hypothesize that inflammatory responses, mitochondrial dysfunction, and oxidative stress discussed above may also damage the endothelial cells of glomerular capillaries [41, 43–45], thus leading to urinary protein leakage and decreased glomerular filtration function.

To our knowledge, few studies have collected systemic and ocular factors, such as renal function and AL, while analyzing the molecular etiology of DR. These factors have been independently associated with DR onset and progression [16–19]. Thus, the differentiated sites identified in previous studies might have been confounded by the presence of these factors. This study obtained various systemic and ocular factors from the participants, and most characteristics were well matched in each comparison group. This increased the reliability of our results.

This study had certain limitations. First, since most patients typically develop DR within 17 years of T2DM diagnosis [11], patients diagnosed with T2DM for 20 years without DR were included in the gene-negative control (NDR) group. However, even if the odds are low, these patients may still develop DR in the future. Future longitudinal studies are needed to determine whether these protective factors are still effective when the diabetes duration is over 20 years. Second, the sample size of this study was relatively small, and therefore the results should be treated with care. However, the adopted extreme phenotypic design greatly increases genetic homogeneity, which provides a robust ability to distinguish signals from noise. Despite the relatively small sample size, two sites were successfully replicated in the longitudinal study and were correlated with OCT/A parameters.

## Conclusion

In conclusion, using extreme phenotypes, this study identified a large number of novel methylation modifications of DR in the discovery stage from a cross-sectional study and further validated these results via a longitudinal study. Cg12869254 and cg04026387 were the two sites corresponding to known genes that were successfully replicated, and the degree of methylation at these sites was associated with macular RNFL and VD. These two novel sites may serve as complementary to the known risk factors that contribute to the pathogenesis of DR and as biomarkers to identify diabetic patients at high risk of incident DR. Monitoring these sites may facilitate risk stratification of the diabetic population and offer a potential tool for screening and therapeutic interventions.

## Supplementary Information

**Additional file 1.** Supplementary methods and results.

**Additional file 2.** Supplementary tables and figures.

## Data Availability

Data are available on reasonable request. Any requests for data can be made to the corresponding author and are subject to ethics approval.

## Abbreviations

DR: Diabetic retinopathy
T2DM: Type 2 diabetes mellitus
EWAS: Epigenome-wide association study
OCT/A: Optical coherence tomography/angiography
AL: Axial length
DMS: Differentially methylated CpG site
RNFL: Retinal nerve fiber layer
VD: Vessel density
HbA1c: Glycated hemoglobin
BMI: Body mass index
SCP: Superficial capillary plexus
DCP: Deep capillary plexus
GO: Gene Ontology
KEGG: Kyoto Encyclopedia of Genes and Genomes
OD: Optical density
3′-UTR: 3′-Untranslated region
IGR: Intergenic region

## References

[1] Zheng Y, Ley SH, Hu FB (2018). Global aetiology and epidemiology of type 2 diabetes mellitus and its complications. Nat Rev Endocrinol.

[2] Vujosevic S, Aldington SJ, Silva P (2020). Screening for diabetic retinopathy: new perspectives and challenges. Lancet Diabetes Endocrinol.

[3] Jampol LM, Glassman AR, Sun J (2020). Evaluation and care of patients with diabetic retinopathy. N Engl J Med.

[4] Cho H, Sobrin L (2014). Genetics of diabetic retinopathy. Curr Diabetes Rep.

[5] Reddy MA, Zhang E, Natarajan R (2015). Epigenetic mechanisms in diabetic complications and metabolic memory. Diabetologia.

[6] Corso-Diaz X, Jaeger C, Chaitankar V, Swaroop A (2018). Epigenetic control of gene regulation during development and disease: a view from the retina. Prog Retin Eye Res.

[7] Maghbooli Z, Hossein-Nezhad A, Larijani B, Amini M, Keshtkar A (2015). Global DNA methylation as a possible biomarker for diabetic retinopathy. Diabetes Metab Res Rev.

[8] Li T, Xu Y, Shi Y (2020). Genome-wide analysis of DNA methylation identifies S100A13 as an epigenetic biomarker in individuals with chronic (>/= 30 years) type 2 diabetes without diabetic retinopathy. Clin Epigenetics.

[9] Chen H, Zhang X, Liao N (2020). Identification of NLRP3 inflammation-related gene promoter hypomethylation in diabetic retinopathy. Invest Ophthalmol Vis Sci.

[10] Miao A, Lu J, Wang Y (2020). Identification of the aberrantly methylated differentially expressed genes in proliferative diabetic retinopathy. Exp Eye Res.

[11] Cabrera AP, Monickaraj F, Rangasamy S, Hobbs S, Mcguire P, Das A (2020). Do genomic factors play a role in diabetic retinopathy?. J Clin Med..

[12] Cabrera AP, Mankad RN, Marek L (2020). Genotypes and phenotypes: a search for influential genes in diabetic retinopathy. Int J Mol Sci.

[13] Sharma A, Valle ML, Beveridge C, Liu Y, Sharma S (2019). Unraveling the role of genetics in the pathogenesis of diabetic retinopathy. Eye (Lond).

[14] Kuo JZ, Wong TY, Rotter JI (2014). Challenges in elucidating the genetics of diabetic retinopathy. Jama Ophthalmol.

[15] Shtir C, Aldahmesh MA, Al-Dahmash S (2016). Exome-based case-control association study using extreme phenotype design reveals novel candidates with protective effect in diabetic retinopathy. Hum Genet.

[16] Wang W, He M, Gong X (2020). Association of renal function with retinal vessel density in patients with type 2 diabetes by using swept-source optical coherence tomographic angiography. Br J Ophthalmol.

[17] Hsieh YT, Tsai MJ, Tu ST, Hsieh MC (2018). Association of abnormal renal profiles and proliferative diabetic retinopathy and diabetic macular Edema in an Asian population with type 2 diabetes. Jama Ophthalmol.

[18] Hainsworth DP, Gao X, Bebu I (2021). Refractive error and retinopathy outcomes in type 1 diabetes: the diabetes control and complications trial/epidemiology of diabetes interventions and complications study. Ophthalmology.

[19] Man R, Gan A, Gupta P (2019). Is myopia associated with the incidence and progression of diabetic retinopathy?. Am J Ophthalmol.

[20] Friedman EA, L'Esperance FJ (1980). Diabetic renal-retinal syndrome: the prognosis improves. Arch Intern Med.

[21] Diagnosis and classification of diabetes mellitus. Diabetes Care. 2010;33 Suppl 1:S62-9. 10.2337/dc10-S062 10.2337/dc10-S062 PMC2797383 20042775

[22] Cockcroft DW, Gault MH (1976). Prediction of creatinine clearance from serum creatinine. Nephron.

[23] Jonsson KB, Frydkjaer-Olsen U, Grauslund J (2016). Vascular changes and neurodegeneration in the early stages of diabetic retinopathy: Which comes first?. Ophthalmic Res.

[24] Carpineto P, Toto L, Aloia R (2016). Neuroretinal alterations in the early stages of diabetic retinopathy in patients with type 2 diabetes mellitus. Eye (Lond).

[25] Das A, Rangasamy S, Naymik M (2018). Novel genetic variants in extreme phenotypes of diabetic retinopathy: DRGen study. Invest Ophth Vis Sci.

[26] Ung C, Sanchez AV, Shen L (2017). Whole exome sequencing identification of novel candidate genes in patients with proliferative diabetic retinopathy. Vis Res.

[27] Emond MJ, Louie T, Emerson J (2012). Exome sequencing of extreme phenotypes identifies DCTN4 as a modifier of chronic Pseudomonas aeruginosa infection in cystic fibrosis. Nat Genet.

[28] Seyres D, Cabassi A, Lambourne JJ (2022). Transcriptional, epigenetic and metabolic signatures in cardiometabolic syndrome defined by extreme phenotypes. Clin Epigenetics.

[29] Shtir C, Aldahmesh MA, Al-Dahmash S (2015). Exome-based case–control association study using extreme phenotype design reveals novel candidates with protective effect in diabetic retinopathy. Hum Genet.

[30] Li D, Lewinger JP, Gauderman WJ, Murcray CE, Conti D (2011). Using extreme phenotype sampling to identify the rare causal variants of quantitative traits in association studies. Genet Epidemiol.

[31] Luo C, Hajkova P, Ecker JR (2018). Dynamic DNA methylation: in the right place at the right time. Science.

[32] Sadhukhan T, Bagh MB, Appu AP (2021). In a mouse model of INCL reduced S-palmitoylation of cytosolic thioesterase APT1 contributes to microglia proliferation and neuroinflammation. J Inherit Metab Dis.

[33] Xu S, Lu J, Shao A, Zhang JH, Zhang J (2020). Glial cells: role of the immune response in ischemic stroke. Front Immunol.

[34] Gong X, Wang W, Xiong K (2022). Associations between peripapillary retinal nerve fiber layer and choroidal thickness with the development and progression of diabetic retinopathy. Invest Ophthalmol Vis Sci.

[35] Fiermonte G, Dolce V, Palmieri L (2001). Identification of the human mitochondrial oxodicarboxylate carrier: bacterial expression, reconstitution, functional characterization, tissue distribution, and chromosomal location. J Biol Chem..

[36] Boczonadi V, King MS, Smith AC (2018). Mitochondrial oxodicarboxylate carrier deficiency is associated with mitochondrial DNA depletion and spinal muscular atrophy-like disease. Genet Med.

[37] Ashraf M, Sampani K, Clermont A (2020). Vascular density of deep, intermediate and superficial vascular plexuses are differentially affected by diabetic retinopathy severity. Invest Ophthalmol Vis Sci.

[38] Grunwald JE, Dupont J, Riva CE (1996). Retinal haemodynamics in patients with early diabetes mellitus. Br J Ophthalmol.

[39] Cringle SJ, Yu DY, Alder VA, Su EN (1993). Retinal blood flow by hydrogen clearance polarography in the streptozotocin-induced diabetic rat. Invest Ophthalmol Vis Sci.

[40] Thomas MC, Brownlee M, Susztak K (2015). Diabetic kidney disease. Nat Rev Dis Primers.

[41] Brownlee M (2001). Biochemistry and molecular cell biology of diabetic complications. Nature.

[42] Hui Z, Chen YM, Gong WK (2022). Shared and specific biological signalling pathways for diabetic retinopathy, peripheral neuropathy and nephropathy by high-throughput sequencing analysis. Diab Vasc Dis Res.

[43] Barrett EJ, Liu Z, Khamaisi M (2017). Diabetic microvascular disease: an endocrine society scientific statement. J Clin Endocrinol Metab.

[44] Forbes JM, Thorburn DR (2018). Mitochondrial dysfunction in diabetic kidney disease. Nat Rev Nephrol.

[45] Reidy K, Kang HM, Hostetter T, Susztak K (2014). Molecular mechanisms of diabetic kidney disease. J Clin Invest.

