# Supplementary material for: Genome-wide DNA methylation analysis of extreme phenotypes in the identification of novel epigenetic modifications in diabetic retinopathy
Source: Clin Epigenetics. 2022 Oct 31;14:137. doi: 10.1186/s13148-022-01354-z (PMC9623976; doi:10.1186/s13148-022-01354-z)
Supplement: Supplementary file 1 — Additional file 1. Supplementary methods and results. [file 13148_2022_1354_MOESM1_ESM.docx]

**Supplementary Materials**

**Supplementary Methods**

The experimental quality was assessed carefully by multiple quality control samples. Sensitivity and efficiency were examined using Staining Controls, which were linked to microbeads covered with dinitrophenol or biotin. The efficiency of single-base extension at the X-staining stage was examined using Extension Controls. Extension Controls consists of hairpin oligos that function as both template and probe, the end of which undergoes single base extension at the 3' end using the probe strand itself as a template during the X-staining phase. Hybridization Controls was used to examine the efficiency of microarray hybridization by using synthetic targets instead of amplified DNA. These targeted synthetic sequences were complementary to the probe on the microarray and provided template for single base extension, and were present in the hybridization buffer at three level: high- (5 pM), medium- (1 pM) and low- (0.2 pM) concentration in this study. Target Removal Controls was used to detected the efficiency of DNA template isolation after the single-base extension phase. Effective target DNA isolation produces very low signal values in the green channel. Bisulfite-Conversion Controls was used to evaluated the conversion efficiency of sulfite on genomic DNA. For Bisulfite-Conversion I, if the sulfite conversion is successful, then the "C" (converted) probe will match and extend the converted sequence. If there is unconverted DNA in the sample, then the "U" (unconverted) probe will be matched and extended. For Bisulfite-Conversion II, if the sulfite conversion reaction is completed, the A base will bind and extend as shown in the red fluorescent channel. If there is unconverted DNA in the samples, the G base will bind to the C base in the unconverted cytosine region, which is shown in the green fluorescence channel. Specificity Controls was used to detect non-specific extension of Infinium I and Infinium II probes. This was mainly for T polymorphic sites. Specificity I was used to detect Infinium I probes. In oligonucleotide design, A/T binds to unmethylated C and G/C binds to methylated C. G/C mismatch controls were used to detect specificity in unmethylated backgrounds where misclassification as methylation occurred. PM (perfect match) controls correspond to a perfect match of A/T and therefore should have high signal values. On the contrary, MM (mismatch) controls correspond to G/C mismatch and therefore should have low signal values. Specificity II was designed to focus on the extension of the Infinium II probes. If the Specificity II probes bind to A at non-polymorphic T sites, fluorescent values will be shown in the red signal channel. On encountering non-specific G bases, there will be signal values in the green channel. The overall performance of the assay was assessed using Non-Polymorphic Controls by querying a particular base in a non-polymorphic region of the human genome. We designed four nucleotides (A, T, G, C), where A and T are detected in the red fluorescence channel and C and G in the green channel. Negative Controls were designed as random sequences that do not hybridize to the target DNA. Negative Controls are important for methylation studies because there will be some degradation of the sequence after sulfite treatment. The mean values of these probe signals were set to be part of the background values. The β value, which is defined as the ratio of methylated probe intensity to overall probe intensity, was used to determine the degree of methylation after normalization using the beta mixture quantile dilation method. The β value was calculated according to the following formula:

$$\beta_{i}=\frac{\max\left( y_{\left( i,methy \right)},0 \right)}{\max\left( y_{\left( i,methy \right)},0 \right)+\max\left( y_{\left( i,unmethy \right)},0 \right)+100}$$

The interval of β value is (0, 1), values closer to 1 indicate higher methylation, and values closer to 0 indicate lower methylation. After acquiring the β value, batch effects were corrected using the champ.runCombat function of the ChAMP package, with the β values being logit transformed.

**Supplementary Results of Quality Control**

Quality control of the staining stage is shown in **Supplementary Figure S1**. In the green channel, only the green signal values were higher and all other signals were within the background values. This is similar to the case of the red channel. This indicated that the staining stage was successful. Quality control of the extension stage is shown in **Supplementary Figure S2**. In the red channel, the red and purple fluorescence signal values (corresponding to A and T bases) were significantly higher than the other fluorescence signal values, while other fluorescence signal values were low. In the green channel, the green and blue fluorescence signal values (corresponding to C and G bases) were significantly higher than the other fluorescence signal values, while other fluorescence signal values were low. This indicates that single-base extension was successful. **Supplementary Figure S3** shows the results of the hybridization stage control. The signals in the red channel were background values, and the signals in the green channel had three distinct gradients within the normal range, indicating that the hybridization stage was successful. The quality control of the DNA template isolation stage is shown in **Supplementary Figure S4.** All signal values in the green channel were below 1000, indicating successful target DNA isolation. The quality control of the conversion efficiency is shown in **Supplementary Figure S5A and S5B.** For Bisulfite-Conversion I, the green channel was used to detect the C1 and C2 controls. These probes exhibited high fluorescence signals, and the signal values of the other probes were within the background values. The red channel was used to detect C3, C4, and C5 controls; therefore, these three probes had high fluorescence signal values, and the signal values of the other probes were within the background values. For Bisulfite-Conversion II, the green fluorescence signal was used to detect unconverted bases; therefore, all probe signal values were within the background signal values. The red fluorescence signal was used to detect the converted bases; therefore, the signal values of all probes were high. This indicated that the conversion of genomic DNA by sulfite was successful. Quality control of non-specific extensions is shown in **Supplementary Figure S6A and S6B**. For Specificity I, in the red channel, the GT(PM) signal values in red were high and the other signal values were within the background signal values, while in the green channel, the GT(PM) signal values in green were high and the other signal values were within the background. For Specificity II, high signal values are shown in the red channel, while all signal values in the green channel are within the background values. This indicated the successful non-specific extension of the probes. **Supplementary Figure S7** shows the overall performance of the microarray using non-polymorphic controls. In the red fluorescence signal channel, the signal values of bases A and T were high, and the remainder of the signal values were low. In the green fluorescence signal channel, the signal values of the C and G bases were high, while the rest of the signals were low. Lastly, the signal values of the negative controls in both the red and green channels were within the background (**Supplementary Figure S8**).

**Supplementary Results of Relationship between Metabolic Indicators and DNA Methylation**

The associations between metabolomic indicators and methylation of these two sites and were also analysis. The degree of methylation of cg04026387 was significantly correlated with higher levels of total cholesterol (β=5.1, 95%CI: 1.8, 8.5; P=0.004), low-density lipoprotein (β=4.2, 95%CI: 1.0, 7.5; P=0.012) and high-density lipoprotein (β=12.9, 95%CI: 5.3, 20.5; P=0.001), after adjusted for covariates (Supplementary Table S4). The association between level of HbA1c and methylation degree of cg04026387 showed borderline statistical significance (β=2.1, 95%CI:-0.1,4.4; P=0.057). On the contrary, levels of triglycerides, uric acid, insulin, and oral antihyperglycemic agents treatment were not associated with the degree of DNA methylation in patients with diabetic retinopathy.
