# Supplementary material for: Genome-wide DNA methylation analysis of extreme phenotypes in the identification of novel epigenetic modifications in diabetic retinopathy
Source: Clin Epigenetics. 2022 Oct 31;14:137. doi: 10.1186/s13148-022-01354-z (PMC9623976; doi:10.1186/s13148-022-01354-z)

**Supplementary Tables and Figures**

[Supplementary Tables 2](#_Toc1276520983)

[Supplementary Table S1 2](#_Toc1126527751)

[Supplementary Table S2 3](#_Toc1336079105)

[Supplementary Table S3 4](#_Toc1392504703)

[Supplementary Table S4 5](#_Toc549758315)

[Supplementary Figures 6](#_Toc1313350811)

[Supplementary Figure S1 6](#_Toc1650156611)

[Supplementary Figure S2 7](#_Toc1578343719)

[Supplementary Figure S3 8](#_Toc1504877489)

[Supplementary Figure S4 9](#_Toc1561046904)

[Supplementary Figure S5 10](#_Toc707600129)

[Supplementary Figure S6 11](#_Toc2018414664)

[Supplementary Figure S7 12](#_Toc1843569836)

[Supplementary Figure S8 13](#_Toc984174736)

[Supplementary Figure S9 14](#_Toc1105738758)

[Supplementary Figure S10 15](#_Toc1975308215)

[Supplementary Figure S11 16](#_Toc1055470532)

[Supplementary Figure S12 17](#_Toc1078307104)

[Supplementary Figure S13 18](#_Toc492999895)

[Supplementary Figure S14 19](#_Toc857325139)

[Supplementary Figure S15 20](#_Toc1595823450)

[Supplementary Figure S16 21](#_Toc1081456767)

[Supplementary Figure S17 22](#_Toc1889778408)

[Supplementary Figure S18 23](#_Toc222564126)

Supplementary Tables

Supplementary Table S1

| **Characteristics** | **Cross-sectional study** | | **Longitudinal study** | **P value** |
| --- | --- | --- | --- | --- |
|  | **DR group** | **Incidence-DR group** | |  |
| Number of subjects | 10 | 10 | | - |
| Age, year | 64.2±3.3 | 66.3±6.4 | | 0.369 |
| Male, % | 6 (60%) | 3 (30%) | | 0.650 |
| BMI, kg/m^2^ | 24.3±2.5 | 26.3±4.2 | | 0.221 |
| SBP, mm Hg | 139.4±22.5 | 139.1±19.4 | | 0.974 |
| DBP, mm Hg | 79.3±13.9 | 70.1±9.5 | | 0.101 |
| HbA1c, % | 6.8±1.5 | 6.9±0.3 | | 0.428 |
| TG, mmol/L | 2.7±2.2 | 2.1±0.9 | | 0.430 |
| TC, mmol/L | 4.7±0.7 | 4.8±0.6 | | 0.777 |
| LDL-c, mmol/L | 2.9±0.8 | 3.1±0.8 | | 0.597 |
| HDL-c, mmol/L | 1.2±0.4 | 1.5±0.3 | | 0.111 |
| Creatinine, μmol/L | 77.1±21.6 | 71.0±14.3 | | 0.347 |
| ACR, mg/g | 10.5±2.1 | 7.3±1.2 | | 0.194 |
| eGFR, mL/min † | 75.2±19.2 | 78.6±6.2 | | 0.568 |
| BCVA, logMAR | 0.2±0.1 | 0.2±0.2 | | 0.397 |
| AL, mm | 23.5±1.5 | 23.3±0.7 | | 0.760 |
| IOP, mmHg | 15.7±2.7 | 15.2±2.3 | | 0.668 |
| T2DM duration, year | 2.2±0.4 | 2.3±0.5 | | 0.615 |
| Insulin, % | 1 (10%) | 1 (10%) | | 0.303 |
| Oral antihyperglycemic agents, % | 10 (100%) | 10 (100%) | | 1.000 |

Comparison of demographic and ocular characteristics between case group in cross-sectional study and in longitudinal nested case-control study.

† The eGFR was calculated using Cockcroft-Gault formula.

BMI= body mass index; SBP= systolic blood pressure; DBP= diastolic blood pressure; HbA1c= glycosylated hemoglobin; TG= triglycerides; TC= total cholesterol; LDL-c= low-density lipoprotein cholesterol; HDL-c= high-density lipoprotein cholesterol; MAU= microalbuminuria; BCVA= best corrected visual acuity; AL= axial length; IOP= intraocular pressure; DR= diabetic retinopathy.

**Supplementary Table S2**

Association between methylation degree of cg12869254 with renal function indicators in univariable and multivariable linear regression models.

| **Renal function indexes*** | **Univariable model 1** | | |  | **Multivariable model 2 §** | | |
| --- | --- | --- | --- | --- | --- | --- | --- |
|  | **β** | **95% CI** | **P value †** |  | **β** | **95% CI** | **P value †** |
| Creatinine, μmol/L | 0.2 | (0.01,0.5) | **0.040** |  | 0.2 | (-0.1,0.5) | 0.101 |
| ACR, mg/g | -0.4 | (-1.3,0.6) | 0.448 |  | 0.02 | (-1.4,1.4) | 0.972 |
| eGFR, mL/min ‡ | -0.2 | (-0.4,0.04) | 0.104 |  | -0.2 | (-0.4,-0.01) | **0.038** |

§ Adjusted for age, sex, T2DM duration and HbA1c. * Per percent of methylation change. † Bold indicates statistically significant.‡ The eGFR was calculated using Cockcroft-Gault formula.

ACR= albumin creatinine ratio; eGFR= estimated glomerular filtration rate; CI= confidential interval; T2DM= type 2 diabetes mellites; HbA1c= glycated hemoglobin.

**Supplementary Table S3**

Association between methylation degree of cg04026387 with renal function indicators in univariable and multivariable linear regression models.

| **Renal function indexes** * | **Univariable model 1** | | |  | **Multivariable model 2 §** | | |
| --- | --- | --- | --- | --- | --- | --- | --- |
|  | **β** | **95% CI** | **P value †** |  | **β** | **95% CI** | **P value †** |
| Creatinine, μmol/L | 0.04 | (-0.1,0.1) | 0.416 |  | 0.1 | (-0.1,0.2) | 0.506 |
| ACR, mg/g | 0.3 | (-0.1,0.6) | 0.127 |  | 0.7 | (0.2,1.1) | **0.014** |
| eGFR, mL/min ‡ | -0.03 | (-0.1,0.1) | 0.548 |  | -0.05 | (-0.2,0.1) | 0.352 |

§ Adjusted for age, sex, T2DM duration and HbA1c. * Per percent of methylation change. † Bold indicates statistically significant. ‡ The eGFR was calculated using Cockcroft-Gault formula.

ACR= albumin creatinine ratio; eGFR= estimated glomerular filtration rate; CI= confidential interval; T2DM= type 2 diabetes mellites; HbA1c= glycated hemoglobin.

**Supplementary Table S4**

Association between methylation degree of cg12869254 and cg04026387 with metabolic indicators.

| **Metabolic indicators** * | **Cg12869254** † | | |  | **Cg04026387 †** | | |
| --- | --- | --- | --- | --- | --- | --- | --- |
|  | **β** | **95% CI** | **P value §** |  | **β** | **95% CI** | **P value §** |
| TG, mmol/L | -0.3 | (-3.4,2.8) | 0.869 |  | -1.4 | (-3.7,0.9) | 0.223 |
| TC, mmol/L | 1.3 | (-3.7,6.3) | 0.593 |  | 5.1 | (1.8,8.5) | **0.004** |
| LDL-c, mmol/L | 0.4 | (-4.3,5.0) | 0.879 |  | 4.2 | (1.0,7.5) | **0.012** |
| HDL-c, mmol/L | 6.2 | (-5.2,17.7) | 0.277 |  | 12.9 | (5.3,20.5) | **0.001** |
| HbA1c, % | -1.0 | (-4.0,1.9) | 0.472 |  | 2.1 | (-0.1,4.4) | 0.057 |
| UA, μmol/L | -0.004 | (-0.04,0.05) | 0.836 |  | -0.01 | (-0.001,0.02) | 0.387 |

† Adjusted for age, sex, T2DM duration and HbA1c. * Per percent of methylation change. § Bold indicates statistically significant.

TG= triglycerides; TC= total cholesterol; LDL-c= low-density lipoprotein cholesterol; HDL-c= high-density lipoprotein cholesterol; HbA1c= glycated hemoglobin; UA= uric acid; CI= confidential interval; T2DM= type 2 diabetes mellites.

Supplementary Figures

Supplementary Figure S1

Schematic workflow of study design.


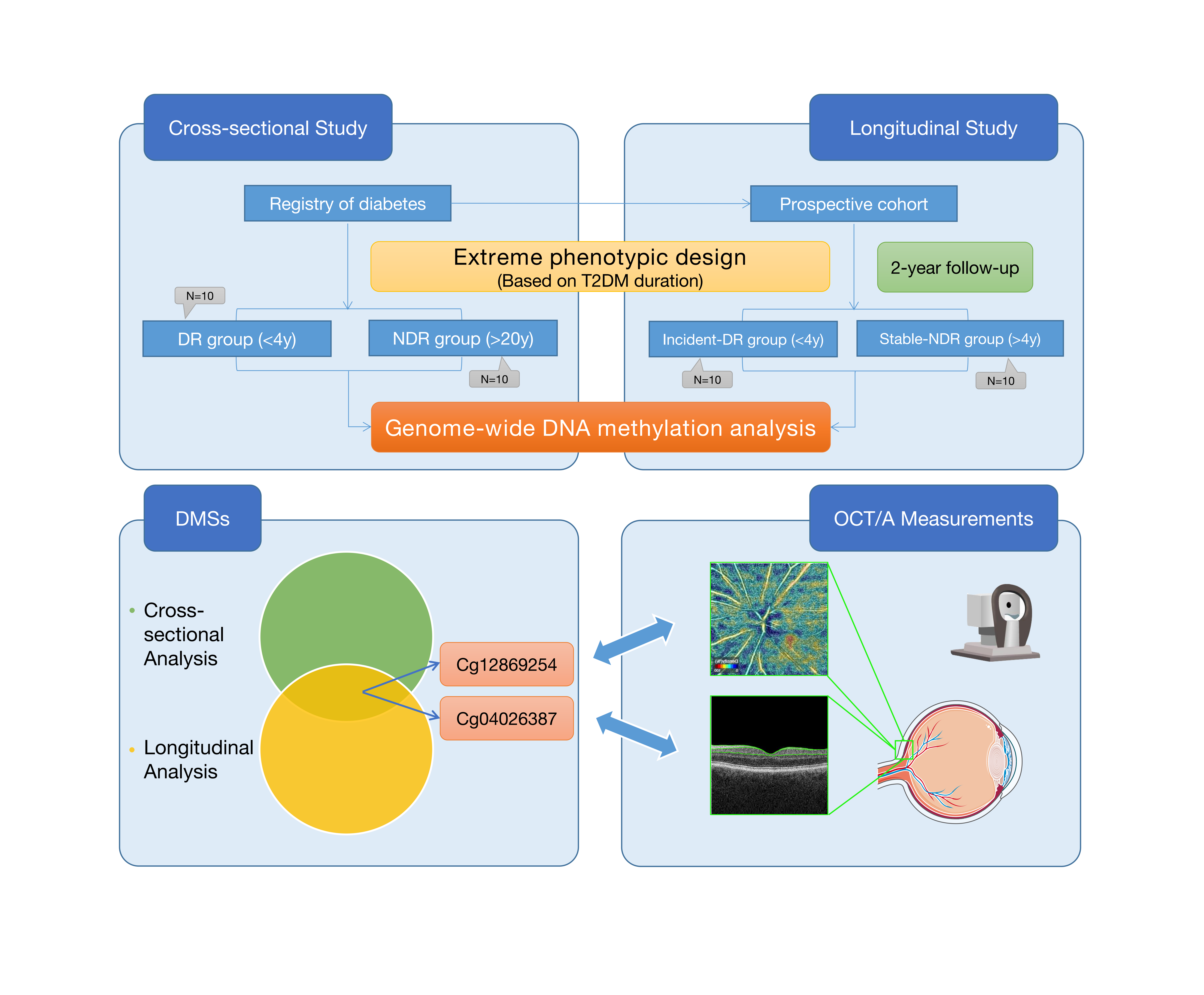


T2DM: type 2 diabetes mellitus; DR: diabetic retinopathy; NDR: non-DR; DMS: differentially methylated CpG site; OCT/A=optical coherence tomography / angiography.

Supplementary Figure S2

Cellular composition of samples from each participants in the cross-sectional study.


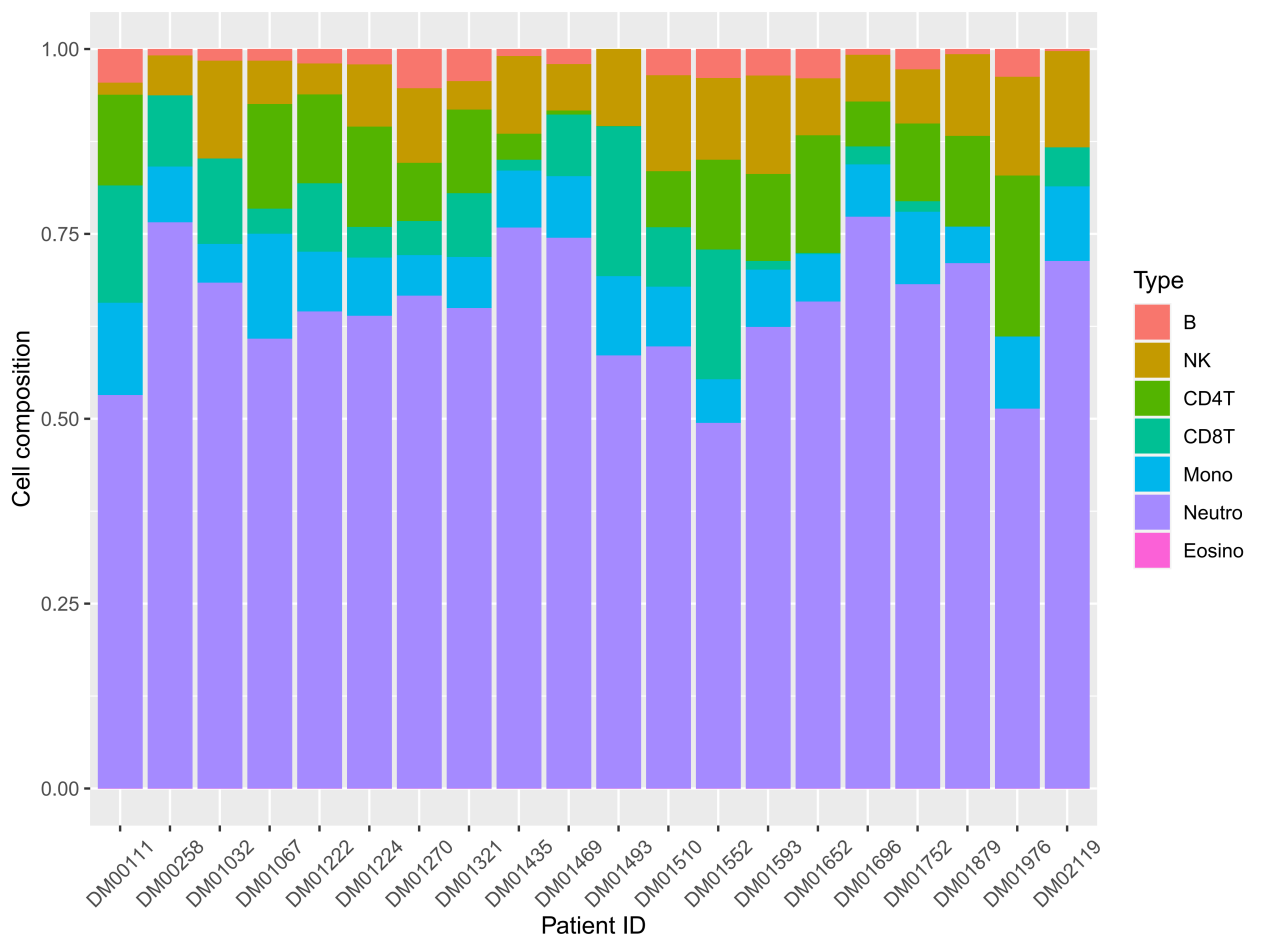


Supplementary Figure S3

Comparison of cellular composition of samples from DR group and NDR group in the cross-sectional study.


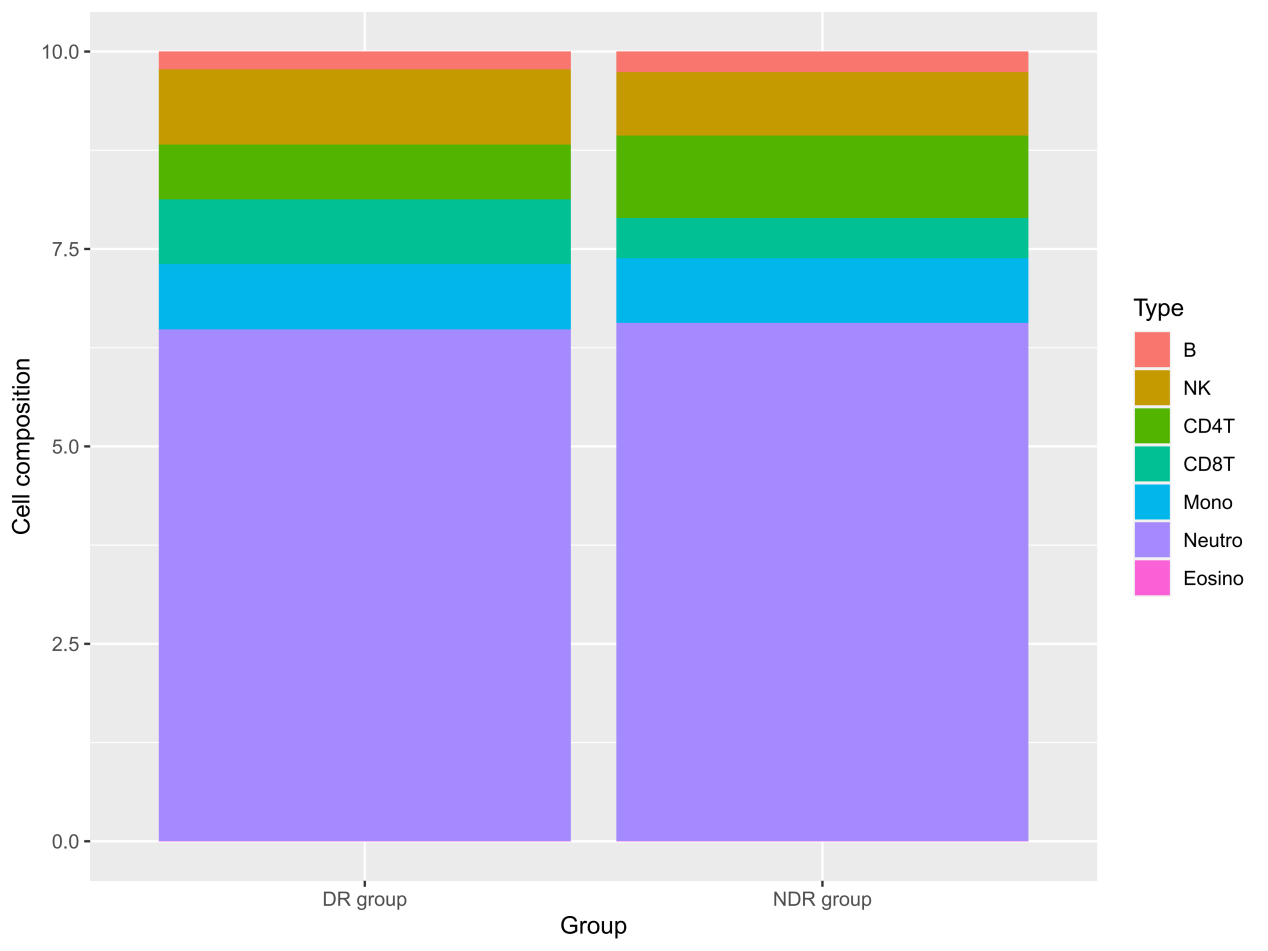


Supplementary Figure S4

Cellular composition of samples from each participants in the longitudinal study.


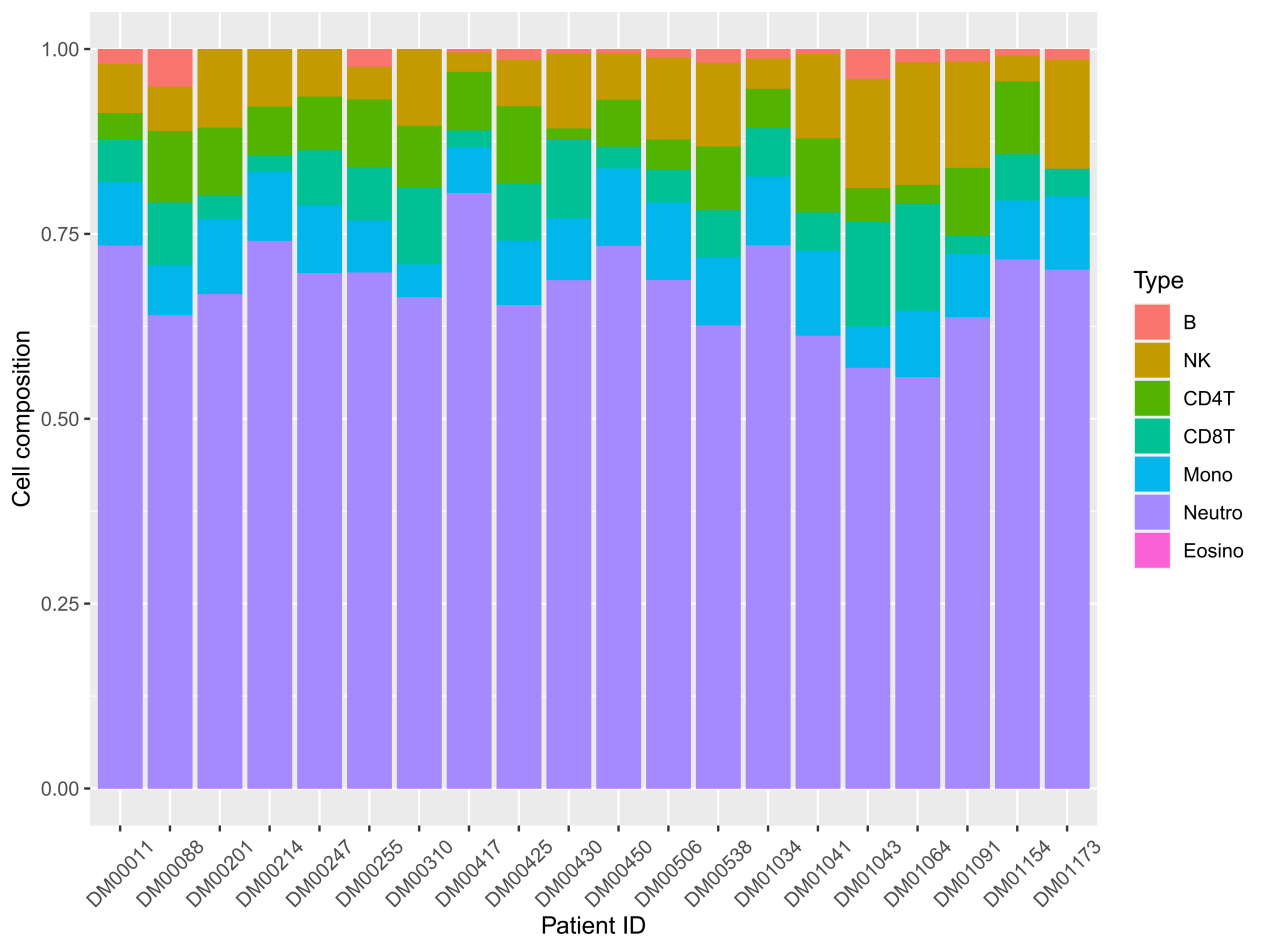


Supplementary Figure S5

Comparison of cellular composition of samples from incidence-DR group and stable-NDR group in the longitudinal study.


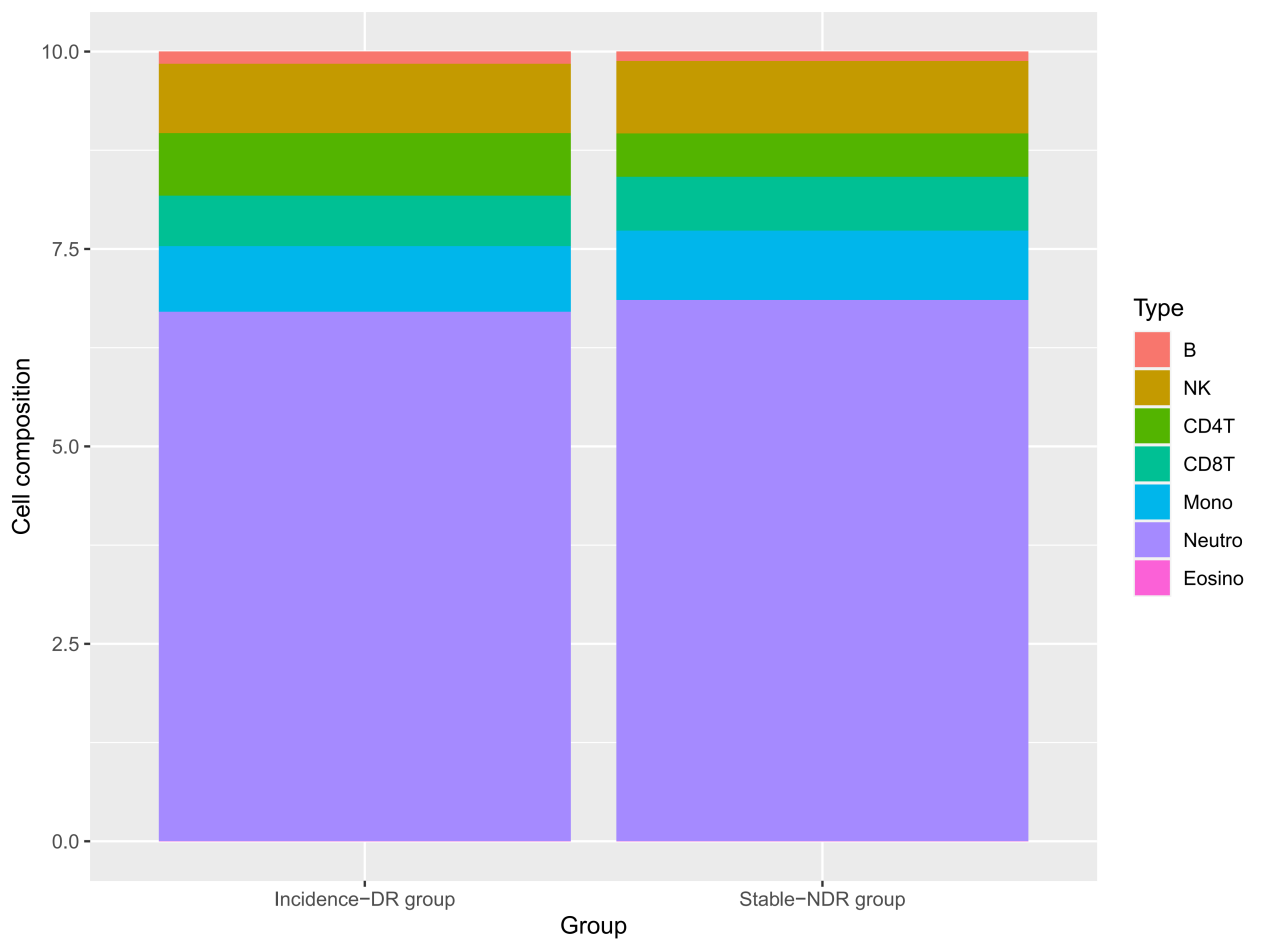


Supplementary Figure S6

Quality control of staining stage. Only high green signal values in green channel and high red signal values in red channel indicate successful staining.


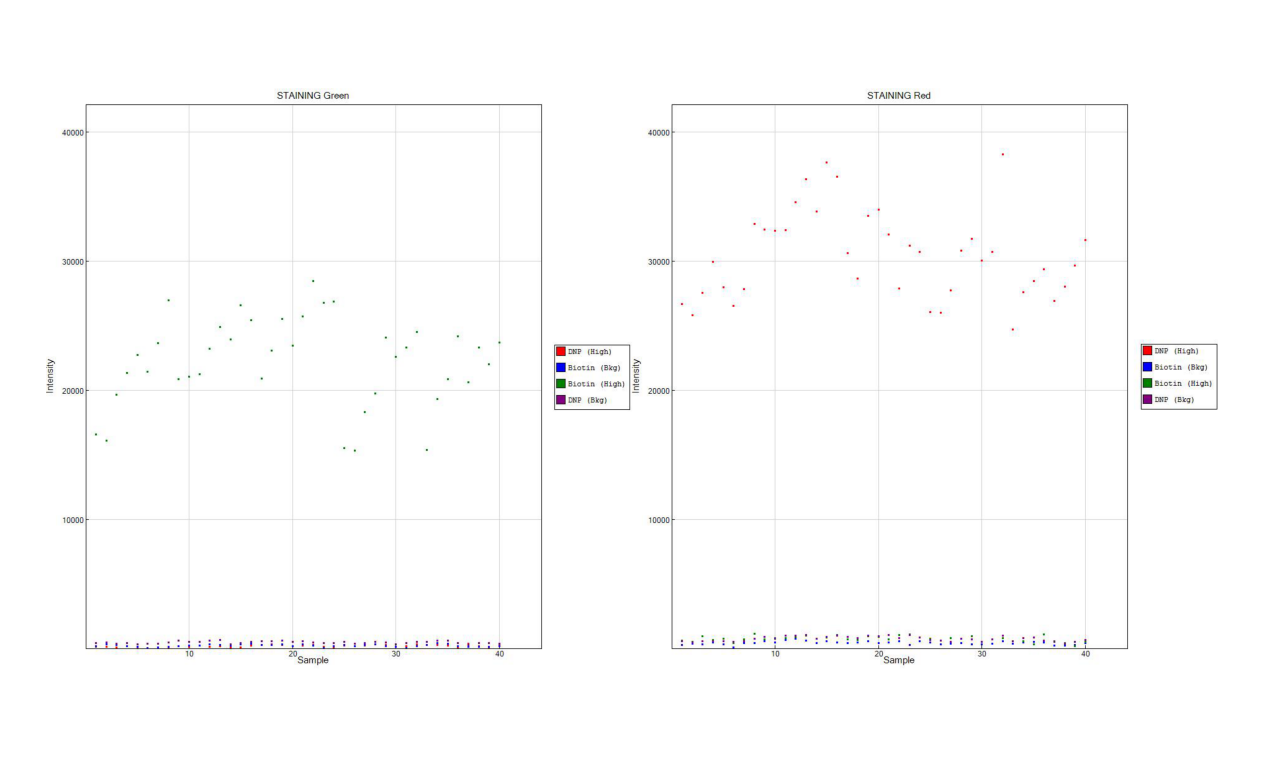


**Supplementary Figure S7**

Quality control of single-base extension stage. Only high green and blue signal values in green channel and high red and purple signal values in red channel indicate successful extension.


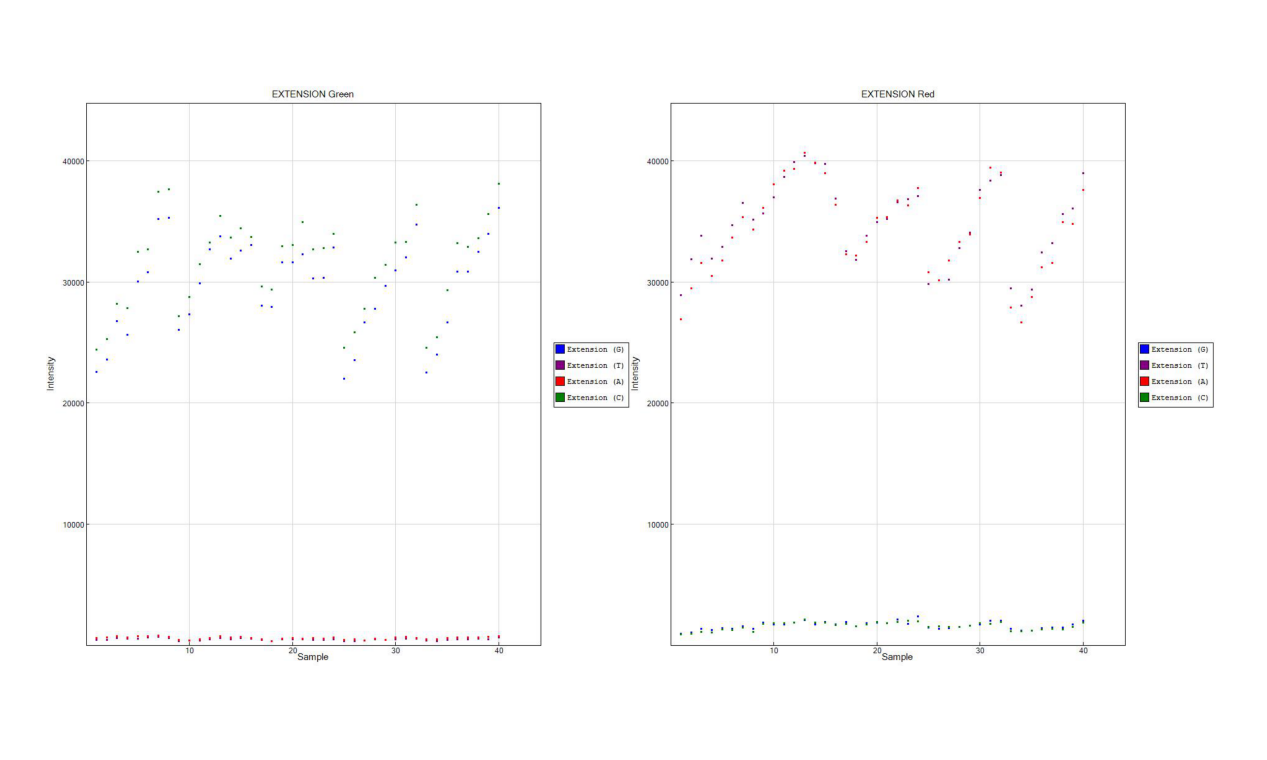


**Supplementary Figure S8**

Quality control of hybridization stage. The signals in the red channel were background values, and the signals in the green channel had three distinct gradients within the normal range, indicating that the hybridization stage was successful.


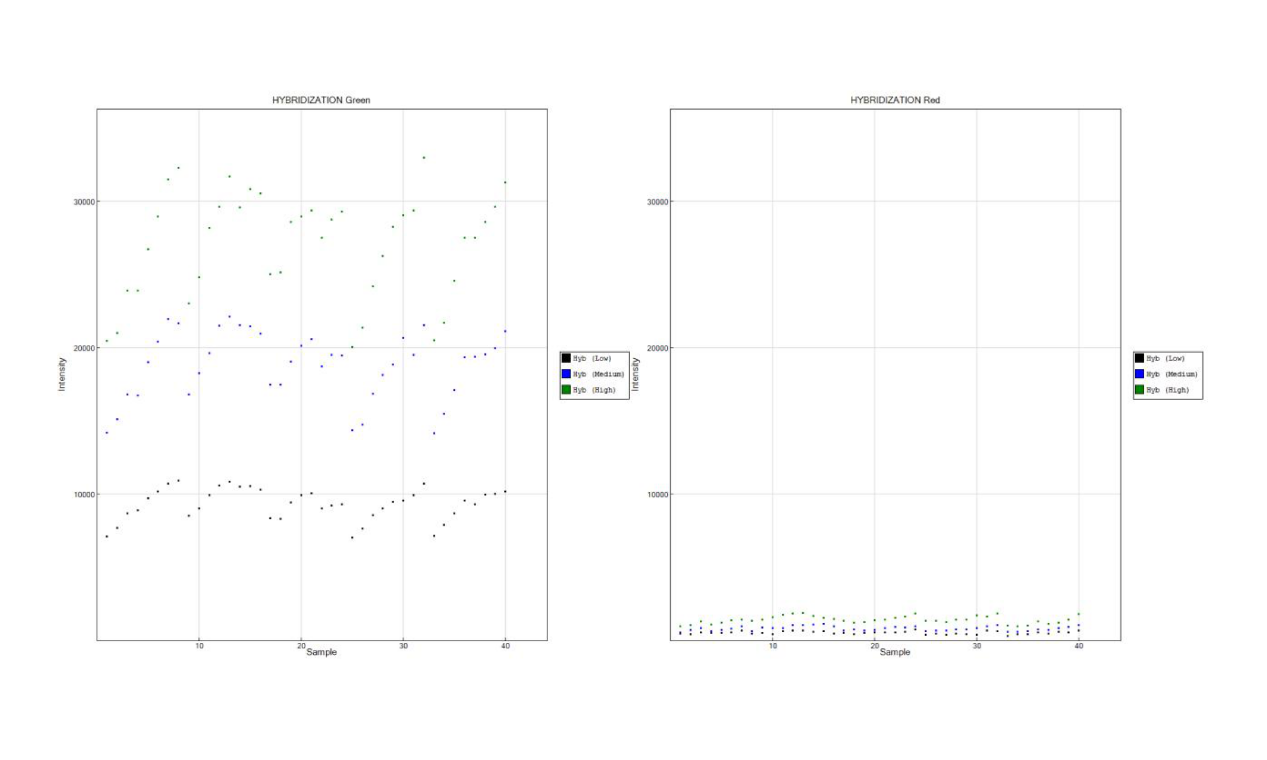


**Supplementary Figure S9**

Quality control of DNA template isolation stage. All signal values in the green channel were below 1000, indicating successful target DNA isolation.


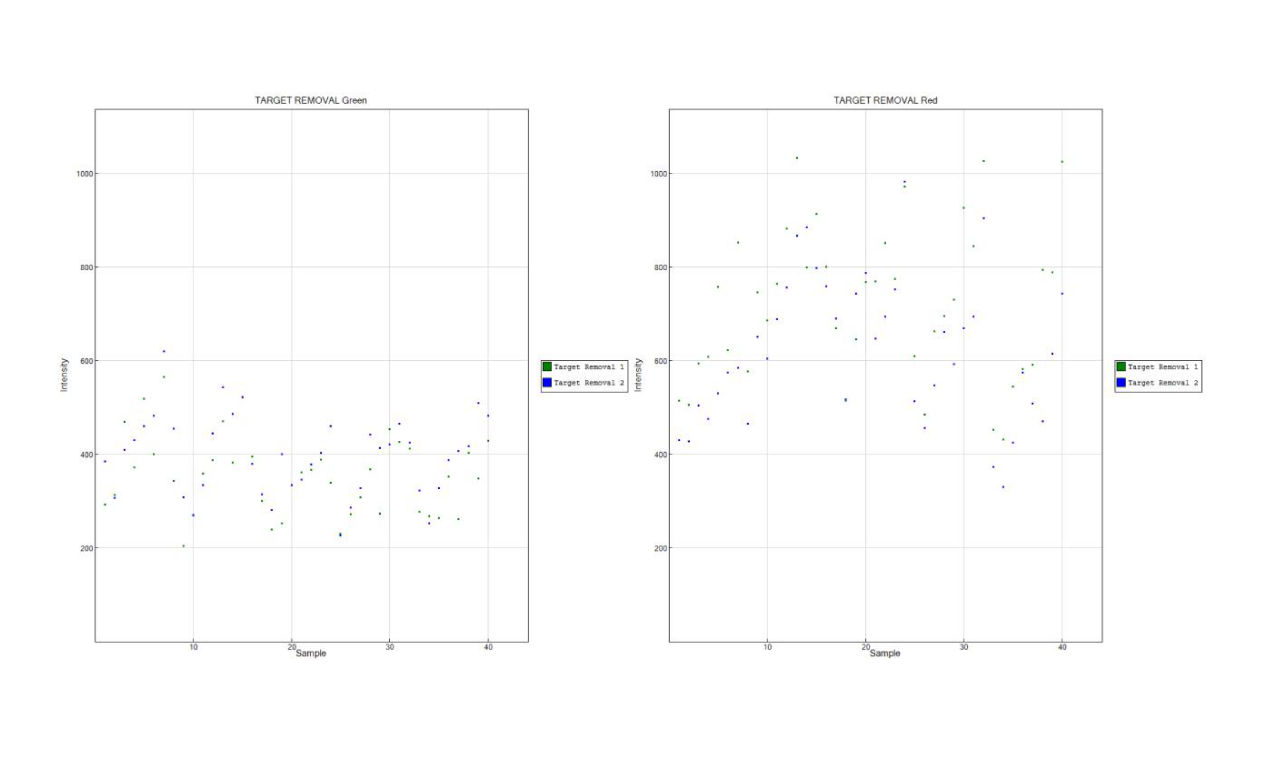


**Supplementary Figure S10**

Quality control of conversion efficiency of sulfite on genomic DNA. For Bisulfite-Conversion I (**A**), C1 and C2 probes corresponded to high fluorescence signals and the signal values of the other probes were within the background values in green channel. C3, C4 and C5 probes had high fluorescence signal values and the signal values of other probes were within the background values in red channel. For Bisulfite-Conversion II (**B**), all probe signal values were within the background signal values in green channel, while signal values of all probes were high in red channel. This indicates that the conversion of genomic DNA by sulfite was successful.


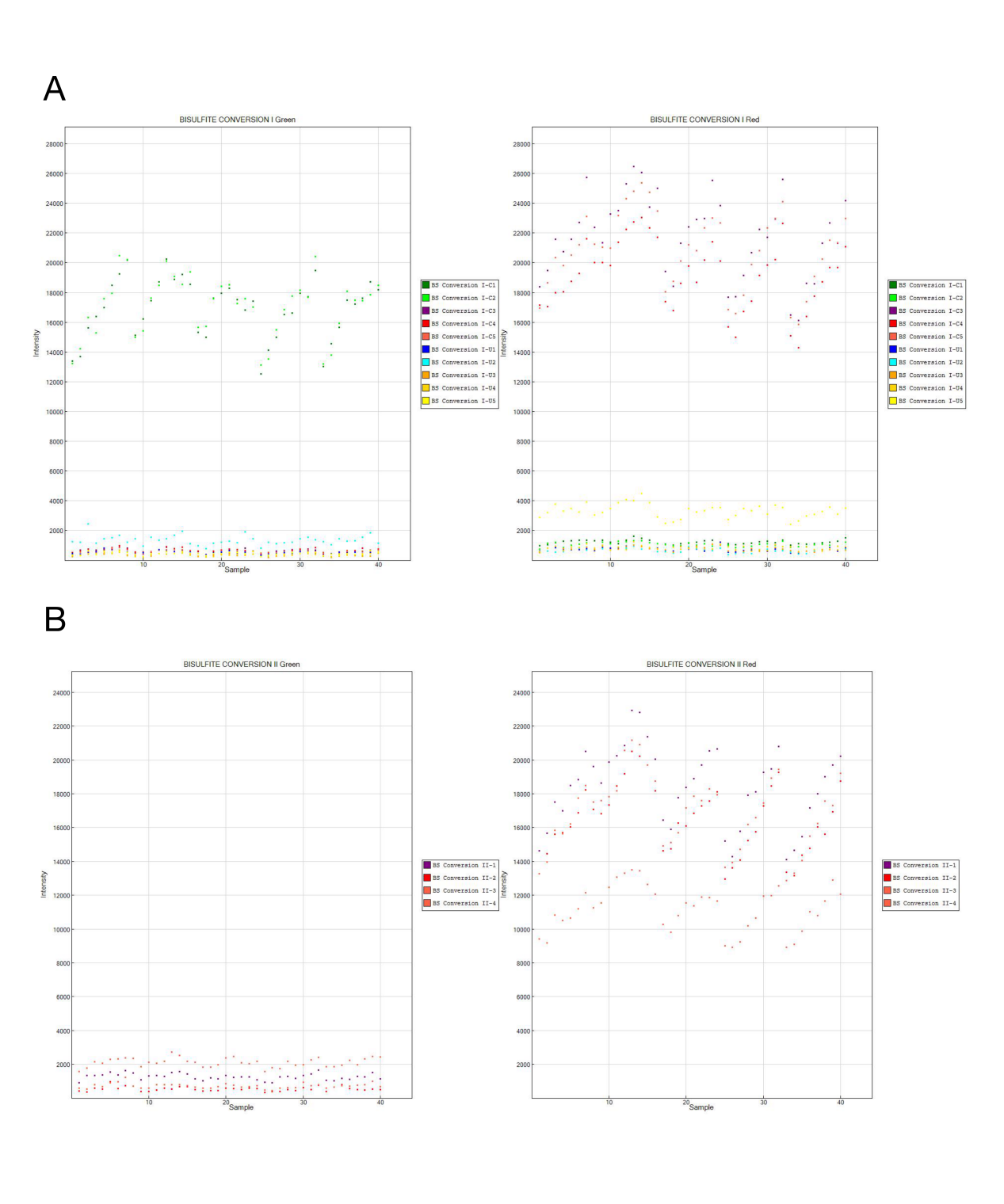


**Supplementary Figure S11**

Quality control of non-specific extension. For Specificity I (**A**), in the red channel, those GT(PM) signal values in red were high and the other signal values were within background signal values, while in the green channel, those GT(PM) signal values in green were high and the other signal values were within the background. For Specificity II (**B**), high signal values were shown in the red channel, while all signal values in the green channel were within background values. This indicates a successful non-specific extension of the probes.


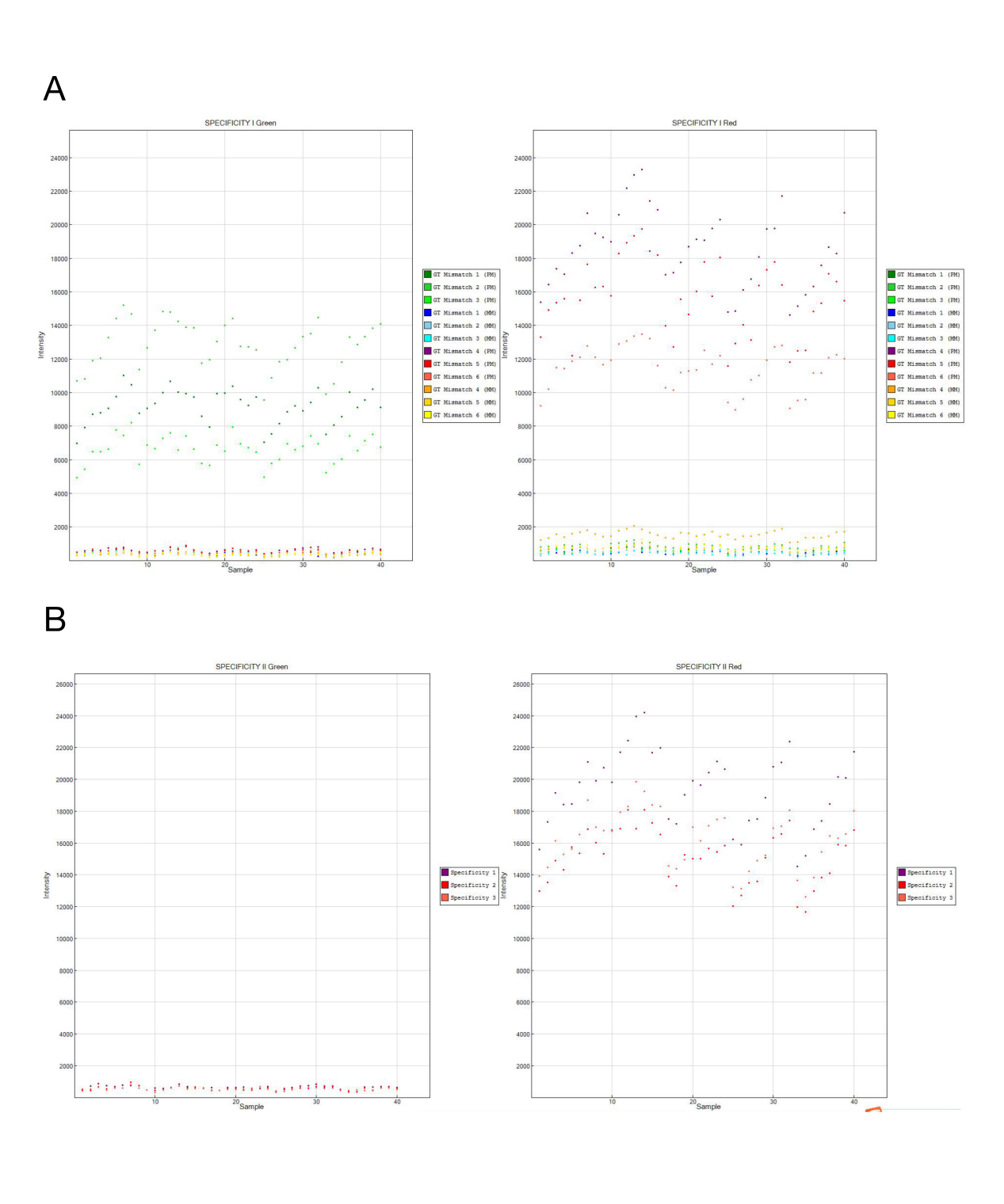


**Supplementary Figure S12**

Quality control of overall performance of the microarray using Non-Polymorphic Controls. In the red fluorescence signal channel, the signal values of A and T bases were high and the rest of the signal values were low. In the green fluorescence signal channel, the signal values of C and G bases were high, and the rest of the signals were low.


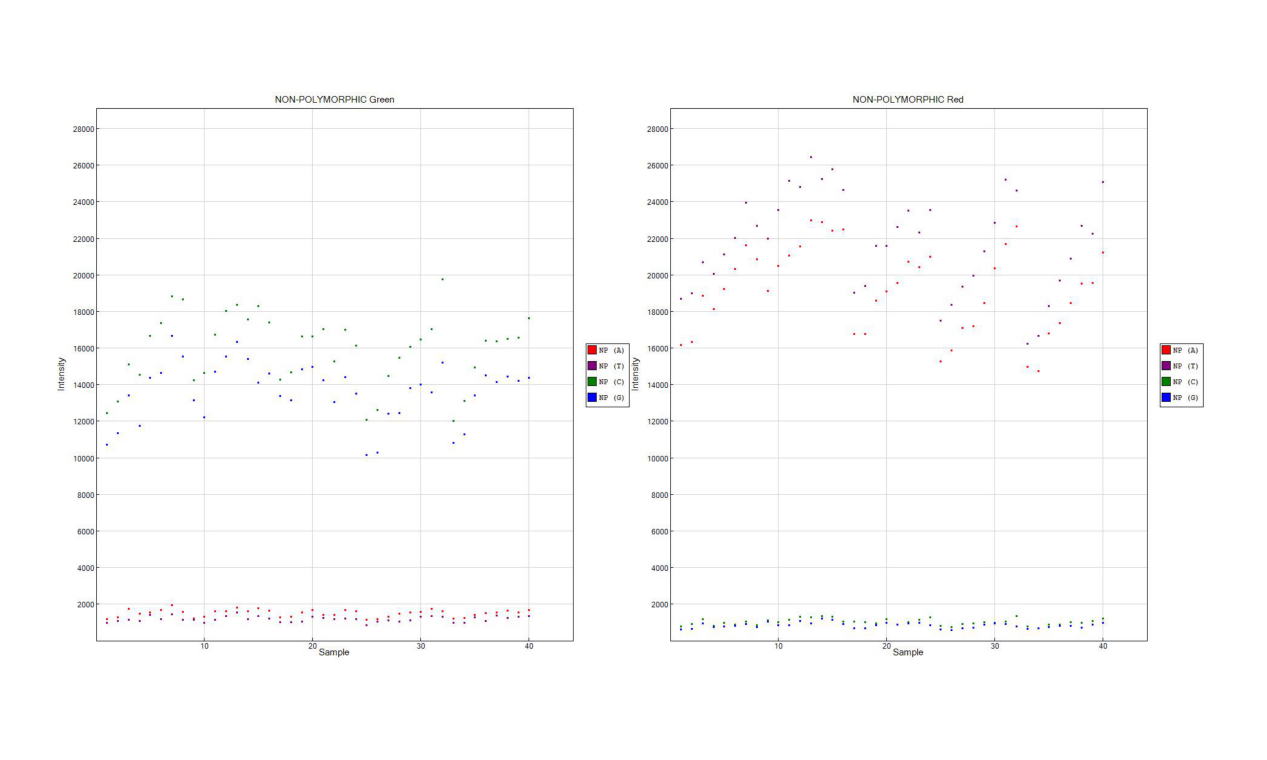


**Supplementary Figure S13**

Negative control of the microarray using random sequences. Signal values of both red and green channels were within background values.


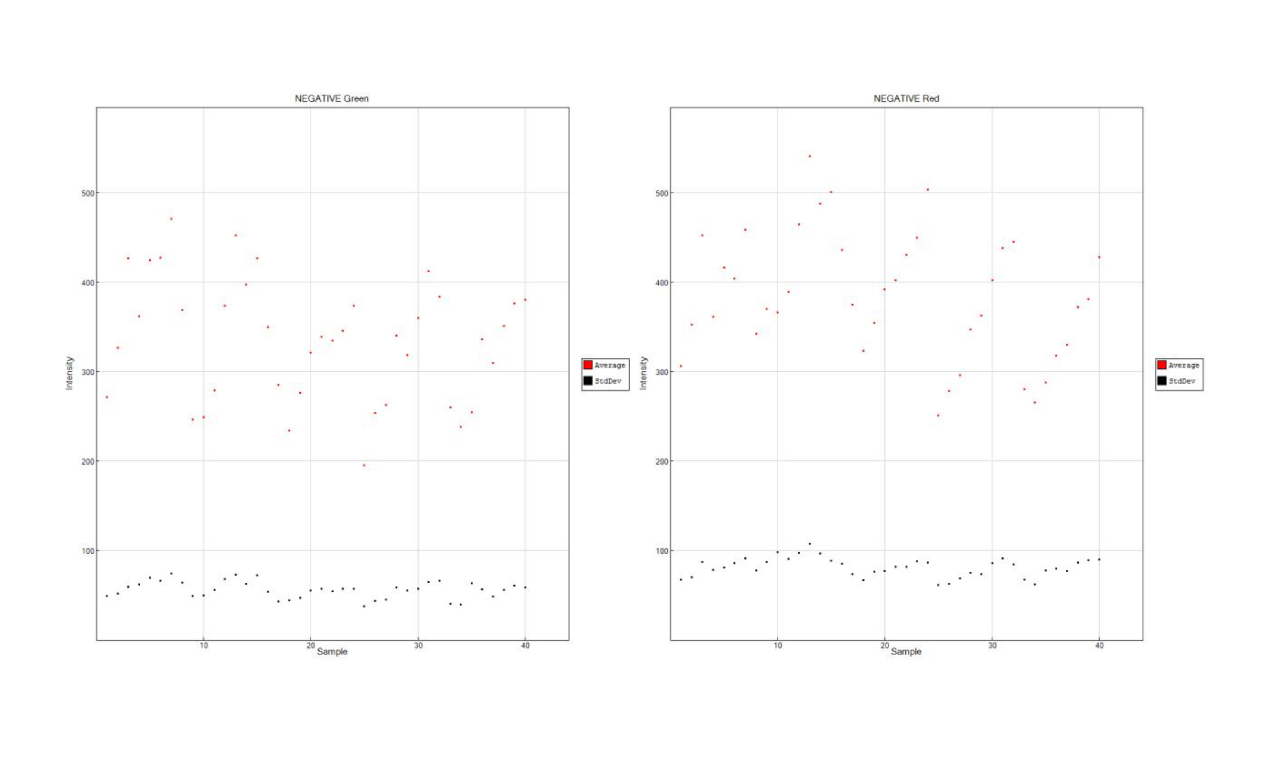


**Supplementary Figure S14**

Distribution of differentially methylated sites of (**A** and **B**) DR group and NDR group, and (**C** and **D**) incidence-DR group and stable-DR group, based on genomic structure domains (**A** and **C**) and regions in relation to nearest CpG island (**B** and **D**).


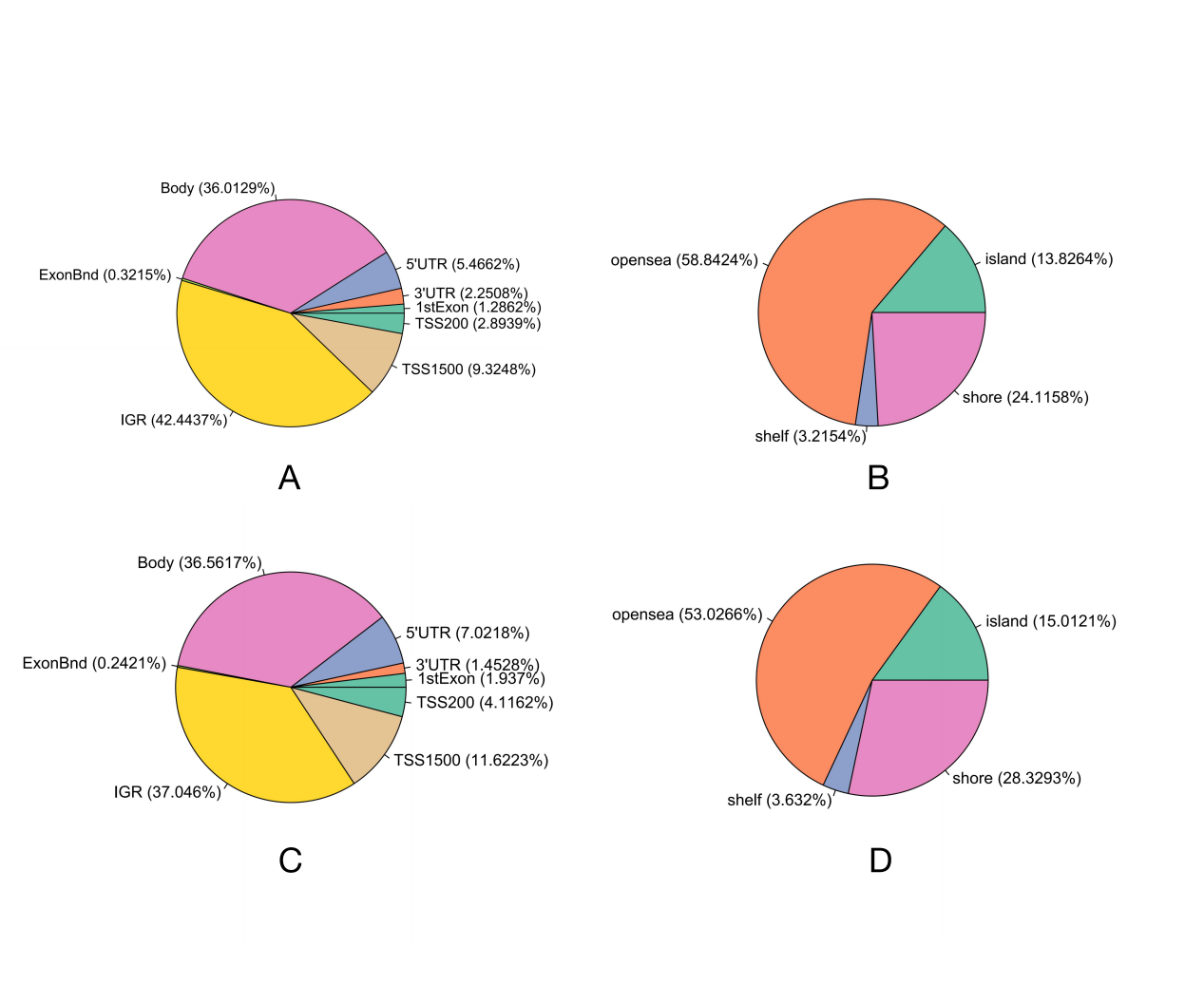


**Supplementary Figure S15**

The GO enrichment analysis of differential methylation sites of (**A**) DR group and NDR group and (**B**) incidence-DR group and stable-DR group. Top 10 most enriched terms in biological process, molecular function and cellular component are displayed respectively in the graph.


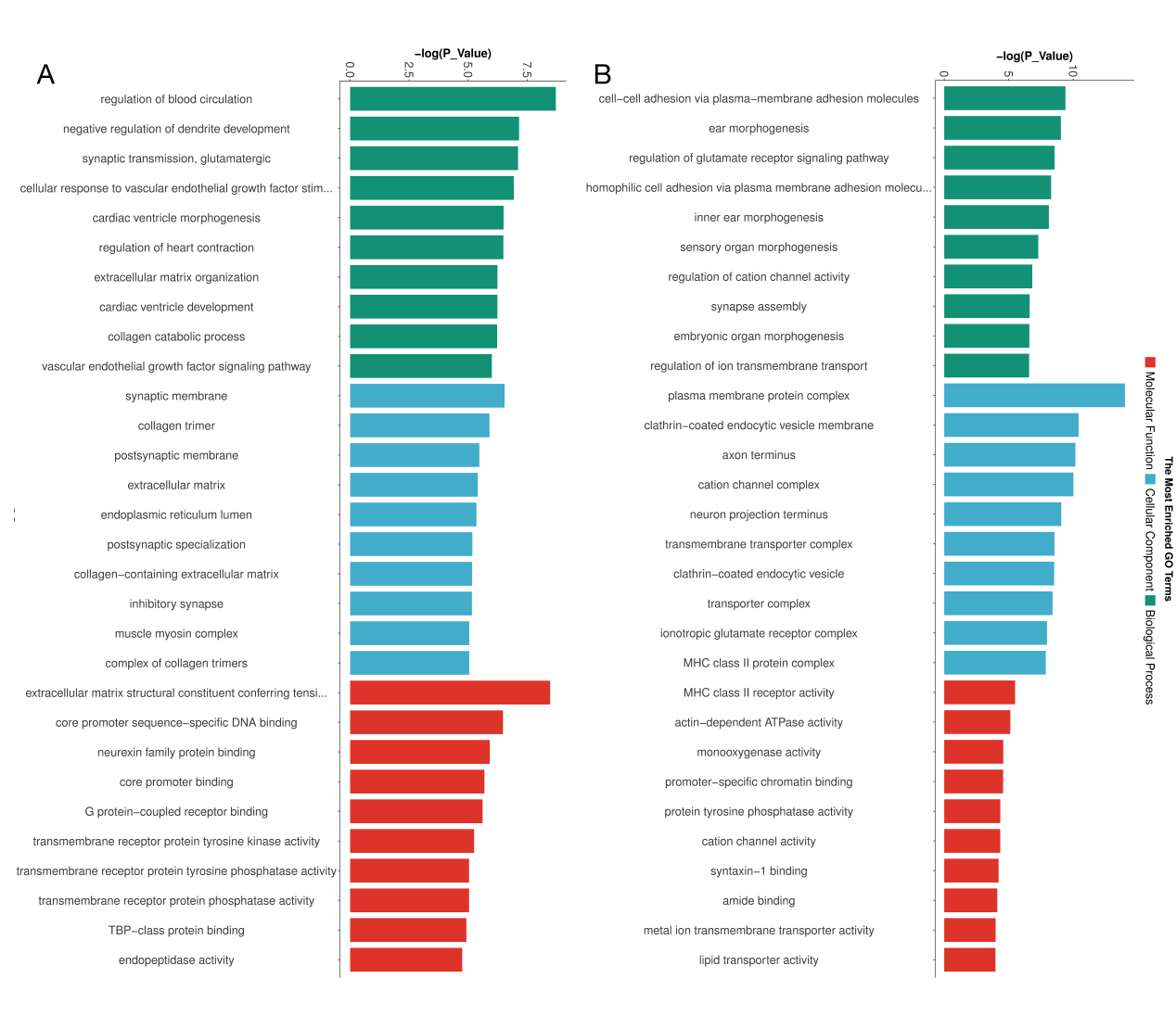


**Supplementary Figure S16**

The KEGG enrichment analysis of differential methylation sites of DR group and NDR group. The size of the circle indicates the number of genes involved, and the color represents the P value.


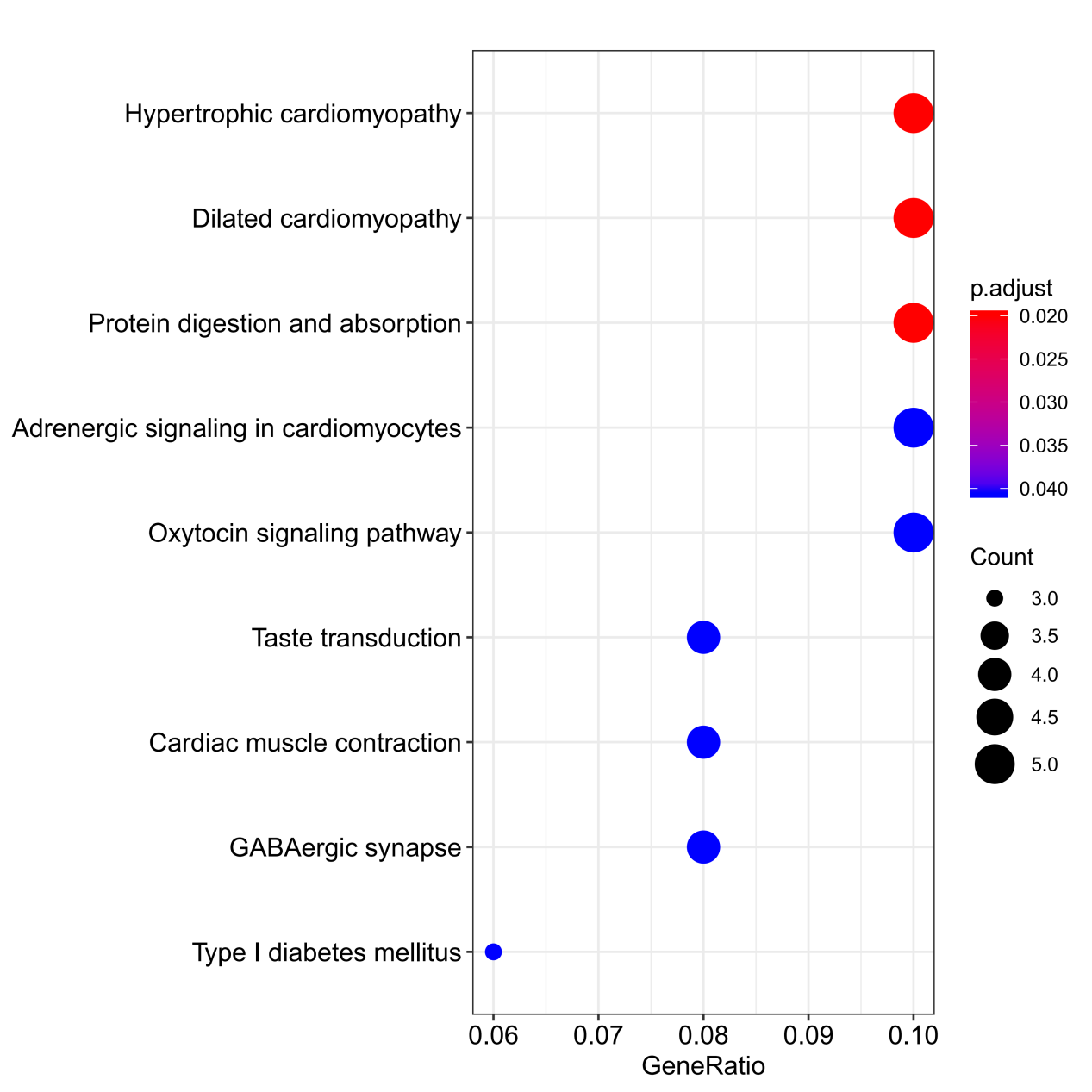


**Supplementary Figure S17**

Manhattan plot for genome-wide DNA methylation analysis in the cross-sectional study.


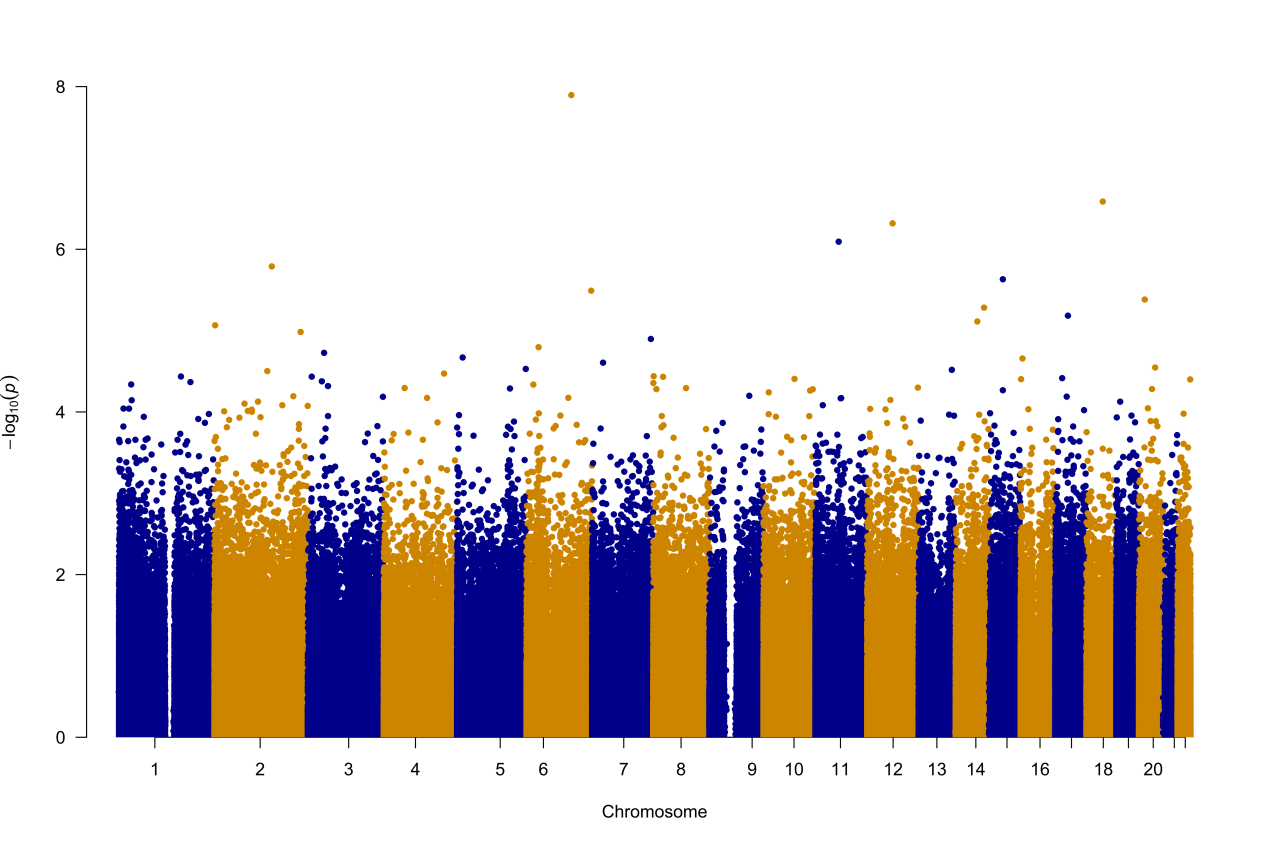


**Supplementary Figure S18**

Manhattan plot for genome-wide DNA methylation analysis in the longitudinal study.


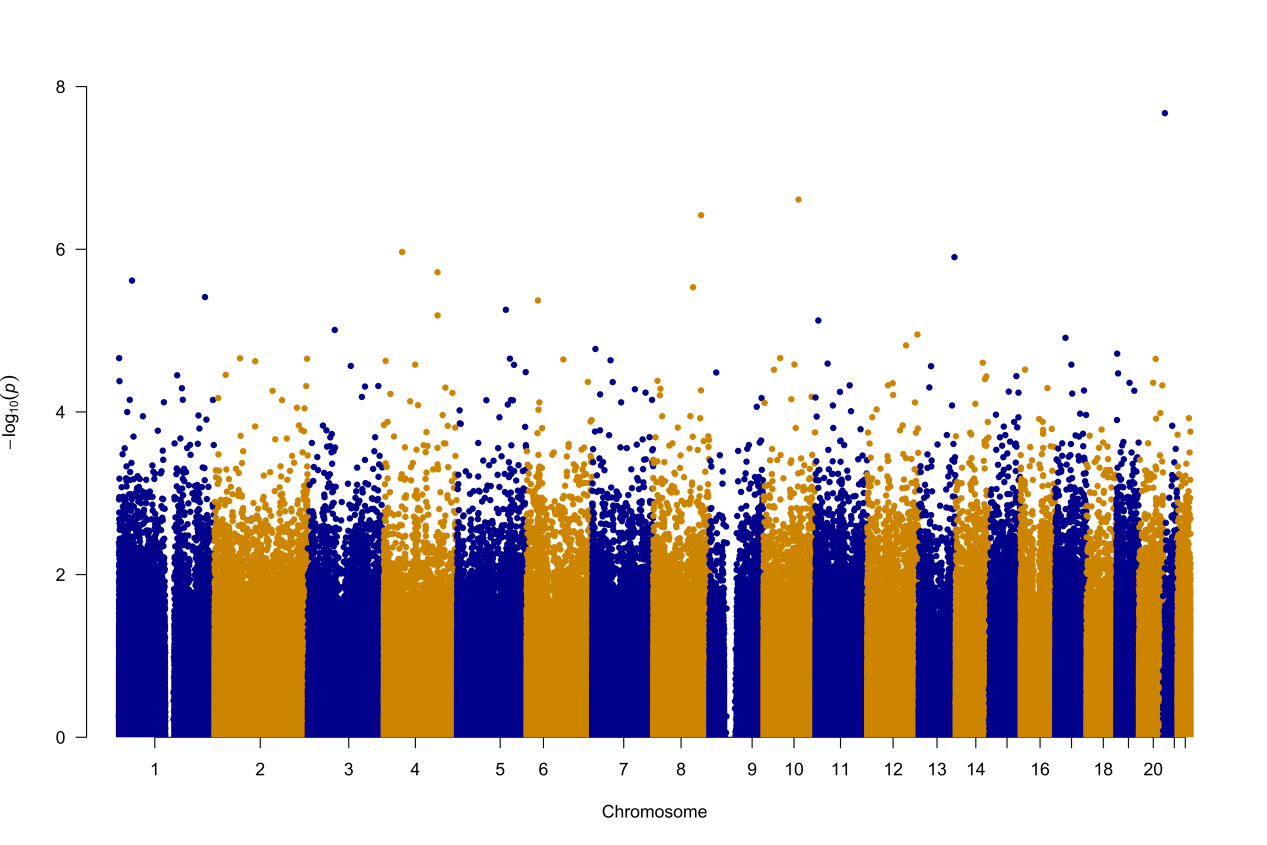

Supplement: Supplementary file 2 — Additional file 2. Supplementary tables and figures. [file 13148_2022_1354_MOESM2_ESM.docx]
